# A multiplex RPA-CRISPR/Cas12a-based POCT technique and its application in human papillomavirus (HPV) typing assay

**DOI:** 10.1186/s11658-024-00548-y

**Published:** 2024-03-08

**Authors:** Yan Liu, Zhujun Chao, Wei Ding, Tanfeng Fang, Xinxian Gu, Man Xue, Wei Wang, Rong Han, Wanping Sun

**Affiliations:** 1 Laboratory of Molecular Diagnostics, College of Pharmaceutical Sciences, Soochow University, Suzhou, 215000 Jiangsu People’s Republic of China; 2 Soochow University, Suzhou Medical College of Soochow University, Suzhou, 215000 Jiangsu People’s Republic of China; 3 Dushu Lake Hospital, Affiliated to Soochow University, Dushu Lake Hospital Affiliated to Soochow University, Suzhou, 215004 Jiangsu People’s Republic of China; 4 Biological Products and Biochemical Drugs, Suzhou Institute for Food and Drug Control, Suzhou, 215101 Jiangsu People’s Republic of China

**Keywords:** HR-HPV, Multiplex RPA, CRISPR/Cas12a, POCT

## Abstract

Persistent infection with high-risk human papillomavirus (HR-HPV) is the primary and initiating factor for cervical cancer. With over 200 identified HPV types, including 14 high-risk types that integrate into the host cervical epithelial cell DNA, early determination of HPV infection type is crucial for effective risk stratification and management. Presently, on-site immediate testing during the HPV screening stage, known as Point of Care Testing (POCT), remains immature, severely limiting the scope and scenarios of HPV screening. This study, guided by the genomic sequence patterns of HPV, established a multiplex recombinase polymerase amplification (RPA) technology based on the concept of “universal primers.” This approach achieved the multiple amplification of RPA, coupled with the CRISPR/Cas12a system serving as a medium for signal amplification and conversion. The study successfully constructed a POCT combined detection system, denoted as H-MRC12a (HPV—Multiple RPA—CRISPR/Cas12a), and applied it to high-risk HPV typing detection. The system accomplished the typing detection of six high-risk HPV types (16, 18, 31, 33, 35, and 45) can be completed within 40 min, and the entire process, from sample loading to result interpretation, can be accomplished within 45 min, with a detection depth reaching 1 copy/μL for each high-risk type. Validation of the H-MRC12a detection system’s reproducibility and specificity was further conducted through QPCR on 34 clinical samples. Additionally, this study explored and optimized the multiplex RPA amplification system and CRISPR system at the molecular mechanism level. Furthermore, the primer design strategy developed in this study offers the potential to enhance the throughput of H-MRC12a detection while ensuring sensitivity, providing a novel research avenue for high-throughput detection in Point-of-Care molecular pathogen studies.

**Supplementary Information:**

The online version contains supplementary material available at 10.1186/s11658-024-00548-y.

## Introduction

Human papillomavirus (HPV) is a non-enveloped, circular, double-stranded DNA virus. High-risk HPV types are detected in 99.7% of cervical cancers, while its low-risk variants primarily cause genital warts or other benign lesions [1]. The World Health Organization's 2020 initiative on cervical cancer prevention and treatment emphasizes that 70% of women should undergo effective cervical cancer screening once before the ages of 35 and 45 [2]. In the latest edition of “Preventing Cervical Cancer: Guidelines for Screening and Treatment of Cervical Pre-Cancerous Lesions” in 2021, HPV DNA testing is recommended as the preferred screening method [3].

**Scheme 1:**
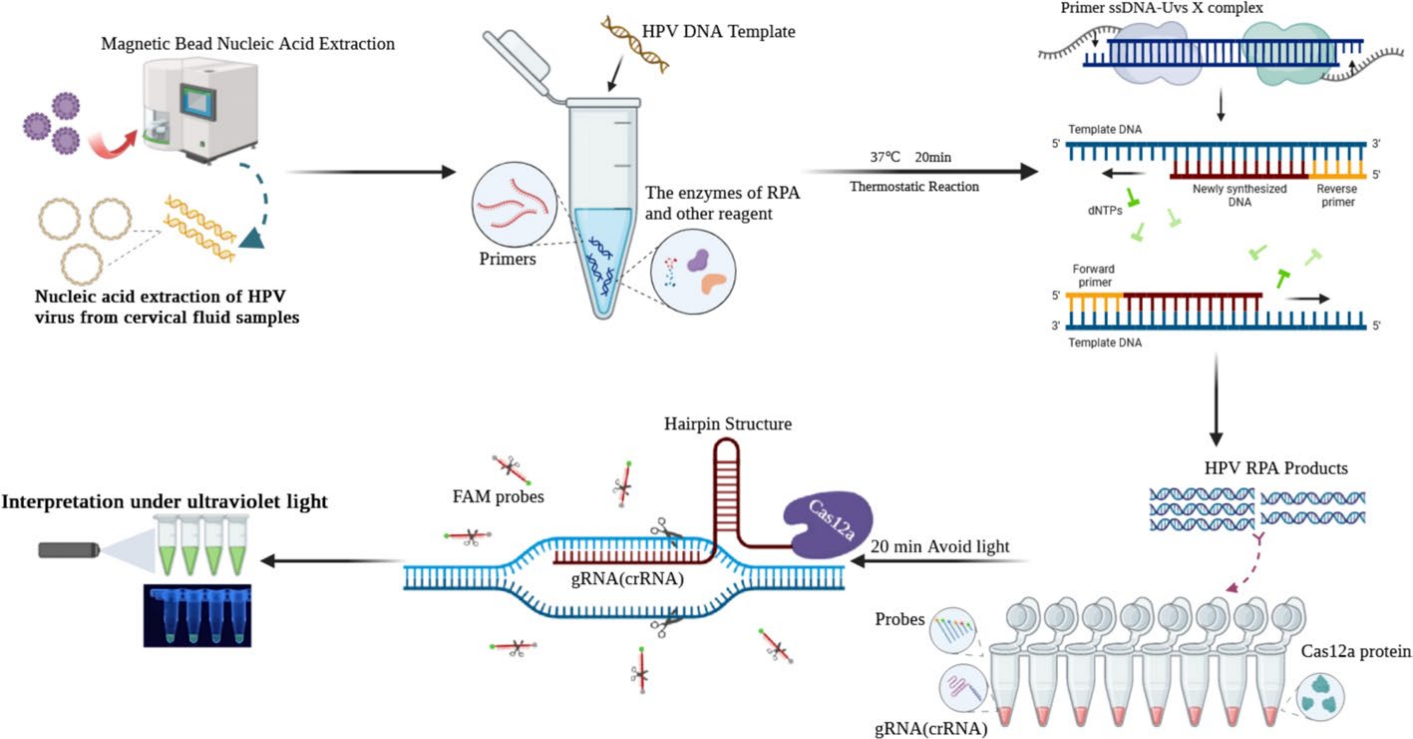
Schematic view of the nucleic acid extraction and HPV-Multiple RPA-CRISPR/Cas12a(H-MRC12a) typing assay

Currently, there is a wide variety of identified HPV types, but only a dozen high-risk HPV types are closely associated with the occurrence and development of gynecological tumors such as cervical and breast cancers. Efficient and sensitive typing detection methods play a crucial role in effectively managing HPV risk stratification. At present, HPV screening methods primarily rely on polymerase chain reaction (PCR) and its derivatives, with QPCR detection results being a key factor in clinical diagnosis [4]. However, these methods are laboratory-based, requiring laboratory instruments and specialized personnel. Their long detection cycles and stringent requirements for testing scenarios make it challenging to implement on a large scale, particularly in regions with limited medical resources. This has resulted in a low prevalence of HPV screening in many parts of the world [5]. Therefore, establishing a point-of-care testing (POCT) for HPV, which does not rely on laboratory equipment and specialized personnel, and even enabling at-home self-testing, could significantly enhance the prevalence of HPV screening. This approach would meet the needs of grassroots medical markets and at-home HPV screening, presenting substantial market potential (Scheme 1).

At present, mature commercialized HPV point-of-care testing (POCT) products, such as HPV test strips, utilize principles related to cytology staining [6]. However, their sensitivity and specificity are significantly lower compared to molecular detection methods. Detection methods based on antibody-antigen interactions have issues with detection window periods and are prone to false positives or negatives. Other ongoing research on HPV nucleic acid POCT detection methods, relying solely on isothermal amplification technologies like LAMP [7], RCA [8] and RAA [9] etc., faces technical limitations such as low detection throughput, an inability to ensure specificity, and a lack of precise typing capabilities, making it challenging to achieve sensitive multiple typing detection. The development of a new generation of HPV POCT detection technology based on molecular principles is urgently needed.

There is a significant amount of research in the field of genetic editing and disease treatment involving the Clustered Regularly Interspaced Short Palindromic Repeats (CRISPR) system. Simultaneously [10–12], interdisciplinary research on its applications has created opportunities for the further development of Point-of-Care (POC) detection technologies based on molecular principles. The artificially editable CRISPR RNA (crRNA) within this system opens up a new avenue for the specific typing detection of the next-generation POC nucleic acid detection methods [13, 14]. Numerous studies have attempted to establish HPV POC detection methods by combining isothermal amplification with CRISPR technology. However, due to common issues associated with multiple amplifications, these methods often focus on only the two major high-risk types, 16 and 18, making it difficult to cover a wide range of high/low-risk types for HPV detection [15, 16]. Those methods that cover a broad spectrum of types may suffer from compromised sensitivity and specificity, or their related validation experiments may not be comprehensive enough [17]. This study employed the combination of Recombinase Polymerase Amplification (RPA) with CRISPR/Cas12a technology to target six major high-risk HPV types: HPV16, 18, 31, 33, 35, and 45. The use of RPA "universal primers" effectively addressed the technical challenge of low amplification throughput caused by primer interactions in multiple amplifications. The study established an amplification system achieving simultaneous amplification of all six types within a single reaction tube. Utilizing the CRISPR/Cas12a system with six crRNAs, the study realized typing detection for these six high-risk HPV types, achieving a detection depth of 1 copy/μL within a reaction time of 40 min at 37 ℃. This detection system can be integrated and consolidated using microfluidic chip technology, allowing for the integration of multiple reaction steps and reagents, thereby facilitating point-of-care (POC) testing for these six high-risk HPV types. Additionally, during the clinical sample validation phase, 34 clinical samples were validated using the QPCR method as a reference. The results demonstrated a high consistency between the constructed H-MRC12a (HPV—Multiple RPA—CRISPR/Cas12a) detection system and the QPCR method. Therefore, this research significantly advances the development of POC high-risk HPV typing detection, promotes the widespread adoption of HPV testing, and contributes to the normalization, precision, and popularization of risk-stratified management for HPV patients.

## Materials and methods

### Main instruments and reagents

PCR Instrument, manufactured by Hangzhou LongGene Scientific Instruments Co., Ltd. Gel Imaging System, manufactured by Hangzhou LongGene Scientific Instruments Co., Ltd. DYY-6C Electrophoresis Apparatus, manufactured by Beijing 61 Instrument Factory. ABI7500 Fluorescent Quantitative PCR Instrument, manufactured by Applied Biosystems, USA. Portable Fluorescent Quantitative PCR Instrument, manufactured by Hangzhou Longji Scientific Instruments Co., Ltd. FAM-Labeled ssDNA Reporter (Fluorescent Dye), purchased from Sangon Biotech (Shanghai) Co., Ltd. TwistAmp Basic Kit RPA Reagents (Lyophilized), purchased from TwistDx, UK. Lba Cas12a, Diluent, 10 × NEB Buffer 2.1, both purchased from New England Biolabs, USA.DreamTaq Green PCR Master Mix, GeneRuler 100 bp DNA ladder, DNA Loading Dye, all purchased from Thermo Fisher Scientific. SuperRed/GelRed Nucleic Acid Stain, purchased from BioSharp Corporation. SSNP-3000A Nucleic Acid Extractor and related extraction reagents, Human Papillomavirus Nucleic Acid Typing Detection Kit (Fluorescent PCR), both purchased from Jiangsu Shuo Shi Biological Technology Co., Ltd.

### Primers, RNA and plasmids

The primers, RNA, and plasmid templates used in this study were all procured from GENEWIZ Biotechnology Co., Ltd. Plasmid concentrations were determined using the Nanodrop 2000/2000C UV–Vis Spectrophotometer, and the plasmids were then gradient-diluted to different concentrations using ultrapure water. The plasmid templates for HPV16, 18, 31, 33, 35, and 45 had sequence lengths of 3224 bp, 3230 bp, 3541 bp, 3419 bp, 3422 bp, and 3424 bp (Additional file 1: Table S1), respectively, with the plasmid itself having a length of 2671 bp.

### Clinical samples and nucleic acid extraction

The clinical cervical fluid samples used in this study were obtained from the gynecology outpatient clinic at the Affiliated Dushu Lake Hospital of Soochow University and the laboratory department of the Affiliated First Hospital of Soochow University. Standard strains were sourced from the Diagnostic Center of the Affiliated Second Hospital of Soochow University. A total of 34 inactivated clinical cervical fluid samples were collected, classified into three types: clear cervical mucus, yellow viscous cervical mucus, and blood-stained cervical mucus. Additionally, three standard strains were collected: Staphylococcus aureus (ATCC 25923), Escherichia coli (ATCC 25922), and Candida albicans (ATCC 14053). Two clinical samples of common gynecological reproductive tract pathogens were also collected: Trichomonas vaginalis and Neisseria gonorrhoeae. Additionally, one serum sample without red blood cells was obtained from peripheral blood. All clinical samples used in this study underwent inactivation treatment as per the hospital’s protocols. The HPV status (positive/negative) of the cervical fluid samples was confirmed by the hospital’s laboratory department. Nucleic acid extraction was performed using a nanomagnetic bead method. This nucleic acid extraction method is simple to operate, easily automated, and holds great potential for point-of-care testing (POCT) development.

### Design of RPA primers and crRNAs

This study adopted the strategy of designing “universal primers” to screen for multiple RPA primers. Using ClustalX software, the full genomes of HPV16, 18, 31, 33, 35, and 45 were aligned. Fragments containing zinc finger protein motifs (CXXC) in the L1 and E6/7 gene regions were prioritized for primer design. The sequence details in the identified regions were observed using SnapGene software, and primer design was performed. The Multiple Primer Analyzer web tool was used for primer editing, CG content adjustment, and primer pair alignment. Sequences with high amplification stability were initially screened. Based on the specific sequences at both ends of this region, refined primer designs were carried out for each type to minimize sequence differences between primers. The optimal primer length was controlled between 28 and 35 nt, with a uniform CG content, and the amplicon length kept within 400 bp. Additionally, the amplicon sequences, excluding the primers, had to contain at least three PAM sites to facilitate subsequent crRNA design and selection. After primer design, the primers were aligned with nucleic acid sequences in the GenBank database using NCBI's BLAST tool. SnapGene software was then used to perform whole-genome sequence alignments with common gynecological reproductive tract pathogens (at least three strains per pathogen subtype) to preliminarily test for specificity. Sensitivity testing was subsequently conducted. In the amplification region selection step, DNA templates were prepared using 10^−1^ ng/μL and 10^−3^ ng/μL concentrations of HPV16 and 18 plasmids (10^7^ and 10^5^ copies/μL). A set of 7 forward primers (HF1–HF7), 8 reverse primers (HR1–HR8), and the corresponding 4 amplification regions were selected for screening (Additional file 1: Table S2). After selecting the amplification regions, refined primer designs were carried out for each HPV type based on the selected region primer combinations (Table 1).

**Table 1:** Sequences of primers

	Primer	Sequences (5′–3′)	Length, nt
Forward	HF5-1-1*	AATAAACCTTATTGGTTACAACGTGCACAGGG	32
	HF5-1-2	AATAAACCTTATTGGTTACAACG**A**GC**G**CAGGG	32
	HF5-1-3*	AATAAACCTTATTGGTTACAACGTGC**T**CAGGG	32
	HF5-2-1	AAT**C**A**G**CC**A**TATTGG**C**TACA**TAAG**GCACAGGG	32
	HF5-2-2*	AAT**C**A**G**CC**A**TATTGG**C**TACA**TAAG**GC**C**CAGGG	32
	HF5-3-1	AATAAACCTTATTGGTT**G**CAACGTGCACA**A**GG	32
	HF5-3-2*	AATAAACCTTATTGGTTACAACGTGCACA**A**GG	32
	HF5-18Y*	ACC**A**TATTGGTTACA**TAAG**GCACAGGG**TCA**	30
Reverse	HR4*	TAAACTGTAAATCATATTCCTCACCATGTC	30
	HR4-1	TAAACTGTAA**T**TCATA**C**TC**T**TCACCATG**C**C	30
	HR4-2	TAAA**T**TGTAAATCATA**C**TCCTC**CA**C**G**TG**C**C	30
	HR4-3	TA**G**ACTGTAAATCATA**C**TC**T**TC**C**CCATG**C**C	30
	HR4-18Q2*	**AA**TAAACTG**C**AAATCATATTCCTCA**A**CATG	30
	HR4-33Q2*	**AAC**AAACTGTA**G**ATCATATTC**T**TCA**A**CATG	30

The above primers were designed with high specificity for various HPV subtypes based on the combination of HF5 and HR4 primers and their corresponding amplification regions. Sensitivity screening was conducted using plasmids of HPV types 16, 18, 31, 33, 35, and 45 as templates. Plasmids for the remaining four HPV types were prepared based on the NCBI HPV full genome sequences (HPV31 reference sequence number LR862053.1; HPV33 reference sequence number LR862077.1; HPV35 reference sequence number LR862022.1; HPV45 reference sequence number LR862061.1); * The primers comprising the final multiplex primer pool

The crRNA in this study consists of a guide sequence and an adjoining RNA sequence. This sequence is closely related to the PAM site. In this study, the commonly used guide sequence GGGAAUUUCUACUGUUGUAGAU [18] was modified by removing the first three thymine bases. The modified guide sequence endowed the gRNA with extremely high base-specific recognition capabilities, a feature later experimentally validated. Based on the autonomously selected multiple RPA system, gRNA designs were conducted according to the PAM sites on the amplicons of various HPV types (Additional file 1: Table S3). The designed crRNA sequences were aligned with the genomic sequences of at least 10 subtypes for each HPV type using SnapGene to ensure their conservativeness. Additionally, NCBI Standard Database alignments were performed to ensure specificity. Finally, the RNA that produced the strongest fluorescence signal was selected through CRISPR reactions to complete the construction of the CRISPR system crRNA pool.

### Construction and optimization of singleplex and multiplex RPA

An experiment was conducted using a 25 μL singleplex RPA reaction system for primer screening. The system components, apart from 15 μL RPA Buffer, included various enzyme functions and other reaction material powders freeze-dried in the tube. Additionally, 1.2 μL each of forward and reverse primers (10 μM), 1.25 μL MgOAc, and 1 μL DNA template were added. The final volume was adjusted to 25 μL by adding nuclease-free water. The system was thoroughly mixed and reacted at 37 °C for 20 min, with shaking initiated at the 4th minute.

Based on the primers selected through the singleplex RPA reaction system, a multiplex RPA reaction system was constructed. The system underwent optimization, including adjustments to the concentration of each primer. The MgOAc dosage was slightly reduced, while the DNA template input was increased to enhance the sensitivity of the multiplex RPA reaction. External stabilizing agents were introduced into the reaction system to improve the homologous recognition specificity of the UvsX recombinase system. The resulting reaction products were purified using a mixture of chloroform and phenol, followed by gel electrophoresis analysis.

### Construction and optimization of CRISPR/Cas12a assay

The selection and construction of the crRNA pool for the CRISPR reaction system were performed using the RPA products as templates. The CRISPR system employed in the screening experiment included Lba Cas12a (1 μM) 2 μL, ssDNA reporter (10 μM) 4 μL, 10 × NEB Buffer 2.1 1 μL, crRNA aqueous solution (10 μM) 0.5 μL, and DNA template 1.5 μL. The reaction system was supplemented with nuclease-free water to a total volume of 12 μL. Multiple-site screening was conducted for crRNAs corresponding to each HPV type to ensure no non-specific cross-reactions or false-positive responses between crRNAs. To maximize the fluorescence endpoint of the CRISPR reaction, the concentrations of Lba Cas12a protein, ssDNA reporter, and crRNA were optimized. A noticeable fluorescence signal in the CRISPR reaction system was observed rapidly after establishing a positive template reaction. During the construction and optimization experiments, the reactions were incubated in a real-time PCR instrument (Q160, Long Gene) for constant-temperature real-time fluorescence monitoring at 37 °C. After 20 min of reaction, fluorescence was immediately observed under UV light, and the fluorescence curves were analyzed.

### Establishment and specificity of H-MRC12a assay

The nucleic acid DNA of the target was introduced into the multiplex RPA reaction system and incubated for 20 min. Subsequently, the RPA amplification solution was added to the CRISPR reaction system and incubated for an additional 20 min in the absence of light, maintaining a constant temperature of 37 °C throughout the entire process. Immediately following completion, fluorescence readings were performed. Leveraging a microfluidic chip and integrated equipment, this combined detection system could accomplish the typing and point-of-care (POC) detection of multiple HPV types within 40 min. The finalized combined reaction system was subjected to specificity validation using genomic DNA from human blood and five common pathogens in the gynecological reproductive tract, including Staphylococcus aureus (ATCC 25923), Escherichia coli (ATCC 25922), Candida albicans (ATCC 14053), Trichomonas vaginalis, and Neisseria gonorrhoeae. The specificity was further confirmed using samples provided by the Affiliated Second Hospital of Soochow University for the three standard strains and samples from the Affiliated Dushu Lake Hospital of Soochow University for the two common gynecological pathogen samples. Additionally, samples of cervical fluid from different types with HPV-negative results, as determined by the hospital, were included for verification.

### Sensitivity of the H-MRC12a assay

The plasmid DNA of HPV16, 18, 31, 33, 35, and 45 was individually gradient-diluted to concentrations of 10^–4^ ng/μL, 10^–5^ ng/μL, 10^–6^ ng/μL, 10^–7^ ng/μL, and 10^–8^ ng/μL (corresponding to 10^4^, 10^3^, 10^2^, 10, and 1 copies/μL). Using the optimized combined reaction system, each concentration of plasmid DNA was subjected to detection. The dual systems were sequentially reacted for 20 min each (totaling 40 min), followed by immediate observation of fluorescence and analysis of the fluorescence signal curves.

### Examination of clinical samples of the H-MRC12a assay and establishment of control methods

The H-MRC12a combined testing system underwent a repeatability and sensitivity assessment using a total of 34 clinical samples with various high/low-risk HPV types. This set included 30 samples with single and double positive results for different HPV types, as well as four negative samples from different types of cervical fluid. Simultaneously, the HPV-positive samples were subjected to a control experiment using the QPCR typing detection method (Shuo Shi Bio HPV nucleic acid typing detection kit—Fluorescent PCR method). The QPCR reaction program included enzyme treatment at 50 °C for 5 min, pre-denaturation at 95 °C for 10 min, denaturation at 95 °C for 10 s, and annealing/extension at 58 °C for 40 s (with 45 cycles of the aforementioned steps, and fluorescence detection at 58 °C). The results obtained from both detection methods were compared to validate the reliability of the H-MRC12a combined testing system.

## Results

### Construction of the multiplex RPA primer pool

**Fig. 1:**
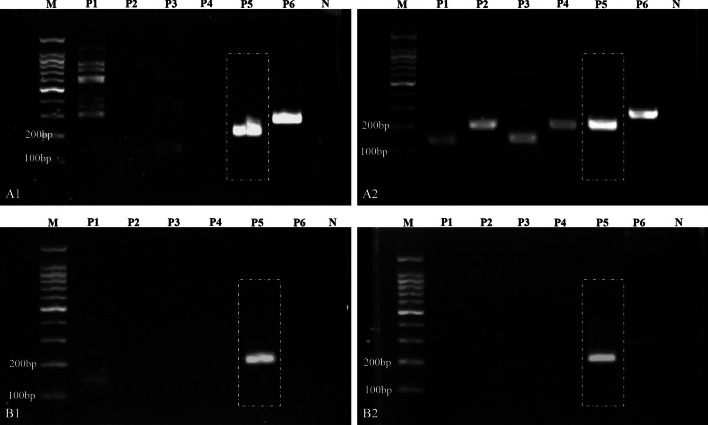
Results of the first round of primer and amplicon region screening. **A1**, **B1** Primer screening results for plasmids of HPV type 16 at concentrations of 10^7^ copies/μL and 10^5^ copies/μL, respectively. **A2**, **B2** Primer screening results for plasmids of HPV type 18 at concentrations of 10^7^ copies/μL and 10^5^ copies/μL, respectively. P1–P6: Primer combinations HF3/HR4, HF3/HR5, HF4/HR4, HF4/HR5, HF5/HR4, HF5/HR5. *N* Negative control. As the exact lengths of the amplicon bands are not yet determined, a separate positive control for this section has not been individually included. The positive results from P1 to P6 can serve as inherent positive controls for this experiment. Gel electrophoresis results indicate that among all designed primer combinations, only the HF5/HR4 combination exhibits higher sensitivity for the amplification of HPV types 16 and 18

**Fig. 2:**
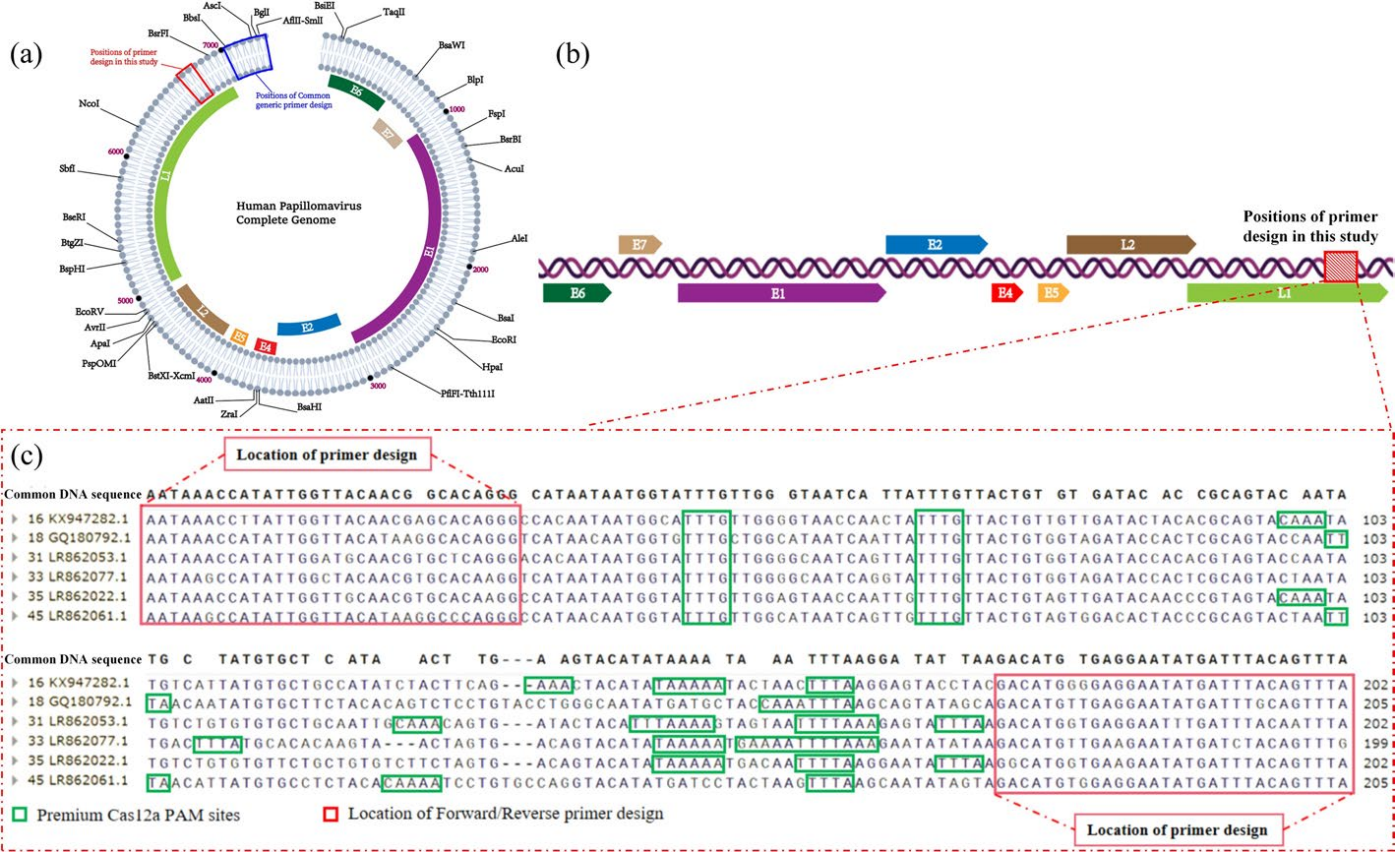
Design positions of multiple RPA primers and crRNA for HPV in this study. **a** Circular representation of the HPV whole genome, with the red box indicating the primer design positions in this study. **b** Schematic representation of the unfolded HPV whole genome. **c** Specific sequences of the 6 HR-HPV amplicon regions and primers along with the PAM sites in this study. The green annotations indicate the positions of PAM sites, while the red annotations indicate the positions of forward/reverse primer designs

**Fig. 3:**
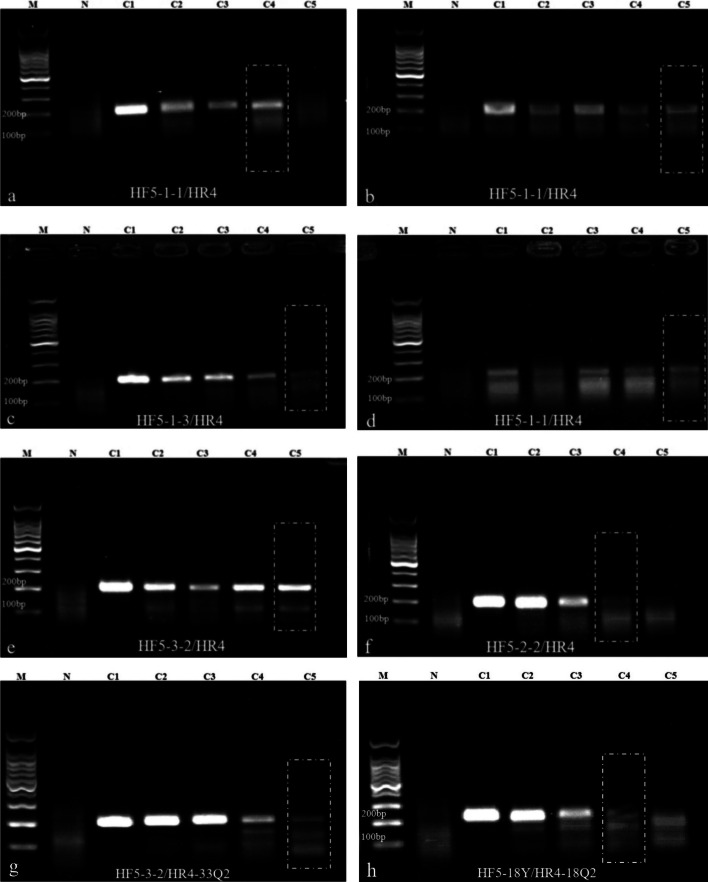
Results of the second round of single RPA primer screening for various HPV subtypes. **a**, **b**, **d** Amplification of plasmids of HPV types 16, 18, and 33 at different concentrations using the HF5-1-1/HR4 primer combination. **c** Amplification of plasmids of HPV type 31 at different concentrations using the HF5-1-3/HR4 primer combination. **e** Amplification of plasmids of HPV type 35 at different concentrations using the HF5-3-2/HR4 primer combination. **f** Amplification of plasmids of HPV type 45 at different concentrations using the HF5-2-2/HR4 primer combination. The forward primers in the above combinations share the same reverse primer, HR4. **g** Amplification of plasmids of HPV type 33 at different concentrations using the HF5-3-2/HR4-33Q2 primer combination. **h** Amplification of plasmids of HPV type 18 at different concentrations using the HF5-18Y/HR4-18Q2 primer combination. C1–C5: Plasmid concentrations of 10^4^ copies/μL, 10^3^ copies/μL, 10^2^ copies/μL, 10 copies/μL, and 1 copy/μL respectively. *N* Negative control. The RPA amplification sensitivity for HPV types 18, 31, 33, and 35 is achieved at 1 copy/μL, while for HPV types 16 and 45, it is achieved at 10 copies/μL

In the initial round of primer and amplification region screening experiments, the primer combination HF5/HR4, which exhibited superior amplification efficiency, was selected from numerous combinations (Fig. 1). The HF5 primer, containing a zinc finger protein-related motif, was found to amplify a stable segment in the posterior section of the L1 region (Fig. 2). This region was confirmed to be stable for amplification across six major HR-HPV types, each containing multiple zinc finger protein motifs, and the amplification efficiency was consistently stable. The length of the amplified fragment for each HPV type ranged from 199 to 205 bp. Building upon this selected primer combination, a detailed design of primers for HPV16, 18, 31, 33, 35, and 45 was carried out based on the specific position in the HPV genome. The high sequence similarity between the upstream and downstream primers in the primer pool minimizes interference among multiple primers, thus enhancing the efficiency of multiplex RPA amplification. In this phase, four forward primers (HF5-1-1, HF5-1-3, HF5-2-2, HF5-3-2) and one reverse primer (HR4) were successfully screened. During the validation experiment for singleplex RPA sensitivity, it was observed that HF5-1-1 effectively amplified HPV16, 18, and 33 types (with sensitivities reaching 1 copy/μL for HPV 18 and 33 types, and 10 copies/μL for HPV 16 type). HF5-1-3 demonstrated effective amplification for HPV31 type (with a sensitivity of 1 copy/μL), HF5-2-2 for HPV45 type (with a sensitivity of 10 copies/μL), and HF5-3-2 for HPV35 type (with a sensitivity of 1 copy/μL) (Fig. 3). These types shared a common reverse primer.

The three primers, HF5-1-1, HF5-1-3 and HF5-3-2, in the multiplex primer pool differ by only a single nucleotide. Three additional specific primers (HF5-18Y, HR4-18Q, HR4-33Q) were added to enhance the amplification efficiency of various HPV fragments. At this stage, it was observed that the forward primer HF5-3-2, when paired with the specific reverse primer HR4-33Q, could effectively amplify HPV 33, with a sensitivity of 1 copy/μL (Table 1, Fig. 3). The final multiplex RPA primer pool comprises five forward primers and three reverse primers, with no primer dimers formed between upstream and downstream primers. The sequence similarity between both forward and reverse primers exceeds 70%.

### Design and screening of crRNAs

**Fig. 4:**
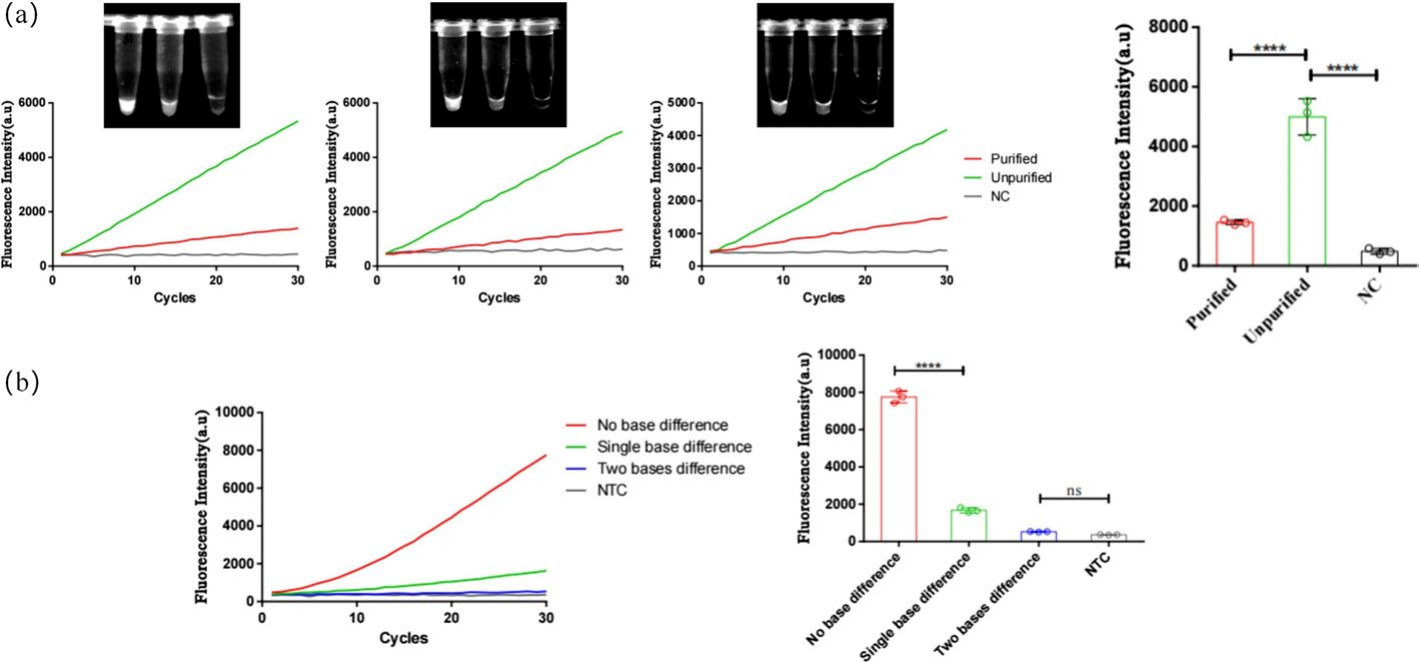
Experimental results exploring the efficiency of primer depletion for Cas12a protein cleavage and validation of structure-specificity in the hairpin structure. **a** Fluorescence curves and endpoint bar charts comparing three parallel CRISPR reactions of RPA products with and without residual primers. Fluorescence was observed immediately under UV light after a 15-min reaction. Purified: RPA products with residual primers removed; Unpurified: RPA products without removing residual primers. **b** Fluorescence curves and endpoint fluorescence values of CRISPR reactions using crRNA with different numbers of mismatched bases compared to the original sequence when connected to a specific hairpin structure. The asterisk indicates significant differences as determined by two-tailed Student’s *t*-test (****P < 0.0001). *ns* denotes no statistical significance. *NC/NTC* negative control reaction

In the construction of the multiplex primer pool, this study took into consideration the design of crRNA. The amplicon lengths for various HPV types ranged from 199 to 205 bp, and all amplicons were located at the same position within the HPV genome. Each HPV type amplicon contained at least five primary Cas12a PAM sites (5′-TTTN or 5′-NAAA) (Fig. 1), providing ample space for crRNA design and selection. The focal point of constructing the CRISPR system lies in the screening of Cas12a PAM sites and the corresponding crRNA design. Although crRNA exhibits high recognition specificity, off-target effects on Cas protein cleavage efficiency can occur when binding to target sequences. The incorporation of a specific bulge structure enables edited crRNA to achieve single-base mismatch recognition, meaning crRNA can recognize target sequences with at most one nucleotide difference. Recognition fails if there is more than one nucleotide difference. To validate the single-base specificity conferred by this bulge structure, a universal crRNA35/45 was designed for HPV 35 and 45 types, each differing by one or two nucleotides from the original sequences. Experimental results demonstrated a significant reduction in the recognition and binding capacity of the guide RNA for sequences with a single nucleotide difference, and almost no recognition for sequences with a double nucleotide difference (Fig. 4b). This confirmed the effective enhancement of recognition specificity and binding stability of crRNA with no nucleotide differences from the original sequence. Ultimately, six crRNAs with lengths ranging from 38 to 43 nucleotides were selected. The addition of different crRNAs in the system allowed for HPV genotyping.

### Establishment and optimization of the multiplex RPA

The construction of a multiplex primer pool involved the amalgamation of HPV type-specific primers identified through single-reaction RPA screening. Initially, the concentrations of individual primers in the mixed primer pool underwent multiple adjustments, with subsequent gel electrophoresis used for result analysis. Adjustment iterations were conducted until plasmids for each HPV type exhibited visible gel bands at a minimum concentration of 10^2^ copies/μL. The adjustment process was terminated upon achieving this threshold. Subsequently, fine-tuning of the magnesium acetate (MgOAc) and DNA template concentrations in the reaction system was undertaken to enhance RPA reaction sensitivity. The MgOAc concentration was ultimately reduced to 1.2 μL, while the DNA template quantity was increased to 2 μL.

**Fig. 5:**
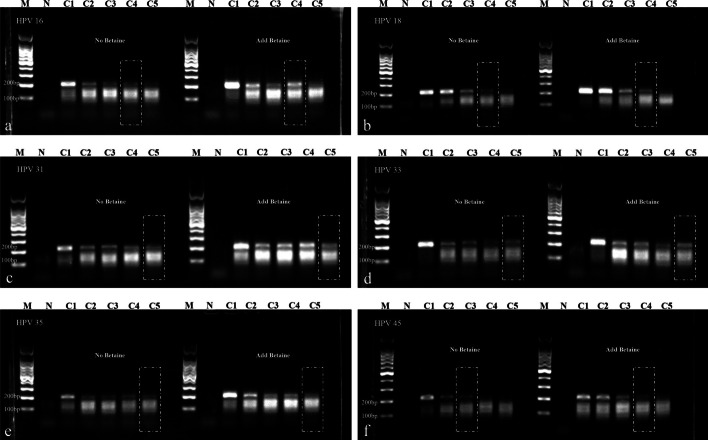
Gel electrophoresis results before and after the addition of betaine in the multiple RPA system. **a**–**f** Gel electrophoresis results of the multiple RPA system amplifying plasmids of HPV 16, 18, 31, 33, 35, and 45 at different concentrations before and after the addition of betaine. C1–C5: Plasmid concentrations of 10^4^ copies/μL, 10^3^ copies/μL, 10^2^ copies/μL, 10 copies/μL, and 1 copy/μL respectively. *N* negative control. Sensitivity for HPV types 16 and 18 reaches 10 copies/μL both before and after the addition of betaine, with brighter bands observed after addition. Sensitivity for HPV types 31, 33, and 35 reaches 1 copy/μL both before and after the addition of betaine, with brighter bands observed after addition. Sensitivity for HPV type 45 surpasses 10^2^ copies/μL after the addition of betaine, reaching 10 copies/μL

Due to the observed substantial decrease in sensitivity for the detection of HPV18 and 33 types in the original multiplex primer pool, a comprehensive reassessment and adjustment of the multiplex primer pool were conducted in this study. Additional modifications included the incorporation of one forward-specific primer (HF5-18Y) and two reverse-specific primers (HR4-18Q, HR4-33Q) into the multiplex primer pool, effectively ameliorating the detection sensitivity for these two HPV types. Furthermore, the introduction of 0.8 M betaine [19] into individual reaction systems was implemented to enhance the sensitivity of the multiplex RPA detection system. Results demonstrated that betaine, through its assisting role on the polymerase and stabilizing effect on protein-ssDNA complexes, significantly improved the amplification efficiency of RPA. This enhancement was evident in the increased brightness of gel electrophoresis bands, and it even facilitated the amplification sensitivity of HPV45 type to surpass 10 copies/μL (Fig. 5). The final establishment of the multiplex RPA system exhibited a detection depth of at least 10 copies/μL for positive plasmids of each HPV type. Notably, HPV 31, 33, and 35 types achieved a sensitivity of 1 copy/μL, while HPV 16, 18, and 45 types achieved a sensitivity of 10 copies/μL. The concentrations of individual primers in the multiplex primer pool were as follows: forward primers HF5-1-1 (264 nM), HF5-1-3 (216 nM), HF5-2–2 (254 nM), HF5-3-2 (216 nM), HF5-18Y (264 nM); reverse primers HR4 (288 nM), HR4-18Q (288 nM), HR4-33Q (288 nM).

In the optimization of the CRISPR system reaction, a considerable amount of residual primers remains after the multiplex RPA reaction. The Cas protein's cleavage targets also include these residual primer ssDNA fragments. To investigate whether the residual primer ssDNA would deplete the cleavage efficiency of the Cas12a protein, thereby reducing the fluorescence signal, a comparative analysis of fluorescence signals was conducted using the same CRISPR reaction system with different amounts of residual RPA reaction products. Since it is challenging to precisely control the concentration of RPA reaction products, only rough estimations within the same order of magnitude were feasible. The products from a single RPA reaction were evenly divided into two portions. One portion underwent direct CRISPR reaction, while the other underwent gel electrophoresis and gel purification to remove primer fragments, with a collection efficiency ranging from 60 to 80%. This approach allowed for the control of the variable of residual primer amounts. Experimental results indicated that the final fluorescence value of the purified product was less than half of the unpurified product (Fig. 4a). This demonstrated that the residual primer amount within the CRISPR system constructed in this study did not significantly affect the reverse cleavage efficiency of the predetermined concentration of Cas12a protein and did not lead to changes in the final detection fluorescence signal intensity.

### Establishment and optimization of the H-MRC12a assay

**Fig. 6:**
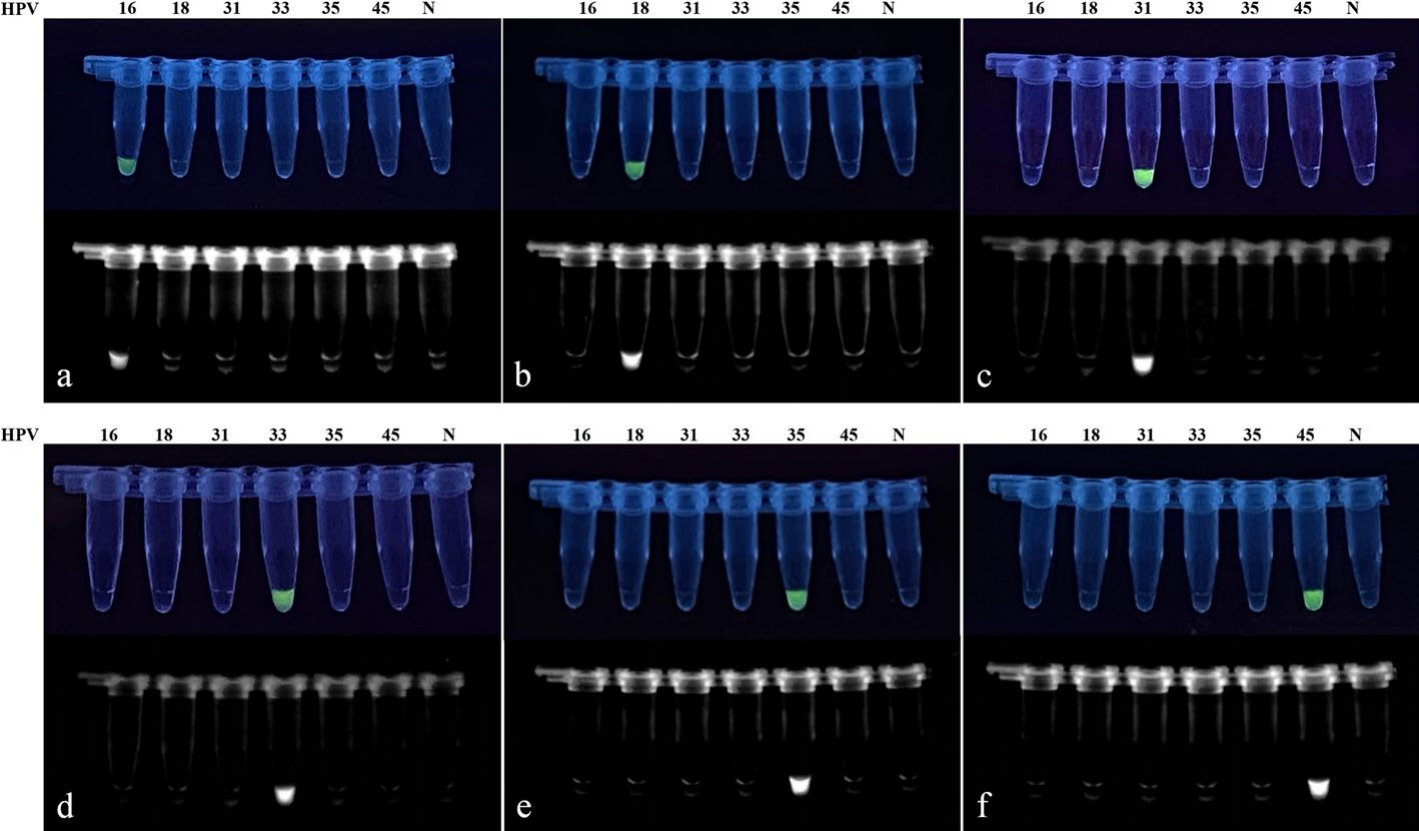
Cross-validation results for the RNA specificity in the crRNA pool of the constructed CRISPR system. **a**–**f** Specificity cross-validation results for CRH1 to CRH6, respectively, against various HPV types. DNA templates for HPV 16–45 were added at concentrations of 10^4^ copies/μL each for the cross-validation. *N* negative control. The experimental results demonstrate that each crRNA shows no cross-reaction with HPV types other than its corresponding type

During the construction of the integrated detection system, this study identified instances where crRNA corresponding to certain PAM sites exhibited non-specific recognition of residual components in the multiplex RPA system, aside from the target sequences. To minimize the risk of false-positive results, an extensive screening of PAM sites was conducted for the six HR-HPV amplicons. After excluding these low-specificity sites, six crRNAs were ultimately selected, demonstrating both specificity and high reverse cleavage efficiency (Table 2). Each crRNA was designed to discriminate a specific high-risk HPV type. Finally, this study assessed the cross-reactivity of each crRNA using high-concentration plasmids representing the six HPV types within the detection spectrum. The results revealed no cross-reaction among the crRNAs (Fig. 6), indicating an orderly and non-interfering typing reaction between them.

**Table 2:** Sequences of crRNAs and the ssDNA Reporter

Oligo Name	Sequences(5′–3′)	Targeted Typing
CRH1	AAUUUCUACUGUUGUAGAUUACUGCGUGUAGUAUCAACAACAG	HPV 16
CRH2	AAUUUCUACUGUUGUAGAUCUGGCAUAAUCAAUUAUUU	HPV 18
CRH3	AAUUUCUACUGUUGUAGAUUUGGGGCAAUCAGUUAUUU	HPV 31
CRH4	AAUUUCUACUGUUGUAGAUUGCACACAAGUAACUAGUGACA	HPV 33
CRH5	AAUUUCUACUGUUGUAGAUUUACUGUAGUUGAUACAAC	HPV 35
CRH6	AAUUUCUACUGUUGUAGAUUUACUGUAGUGGACACUAC	HPV 45
ssDNA reporter	6-FAM-TTTTTTTT-BHQ1 [55]	–

**Fig. 7:**
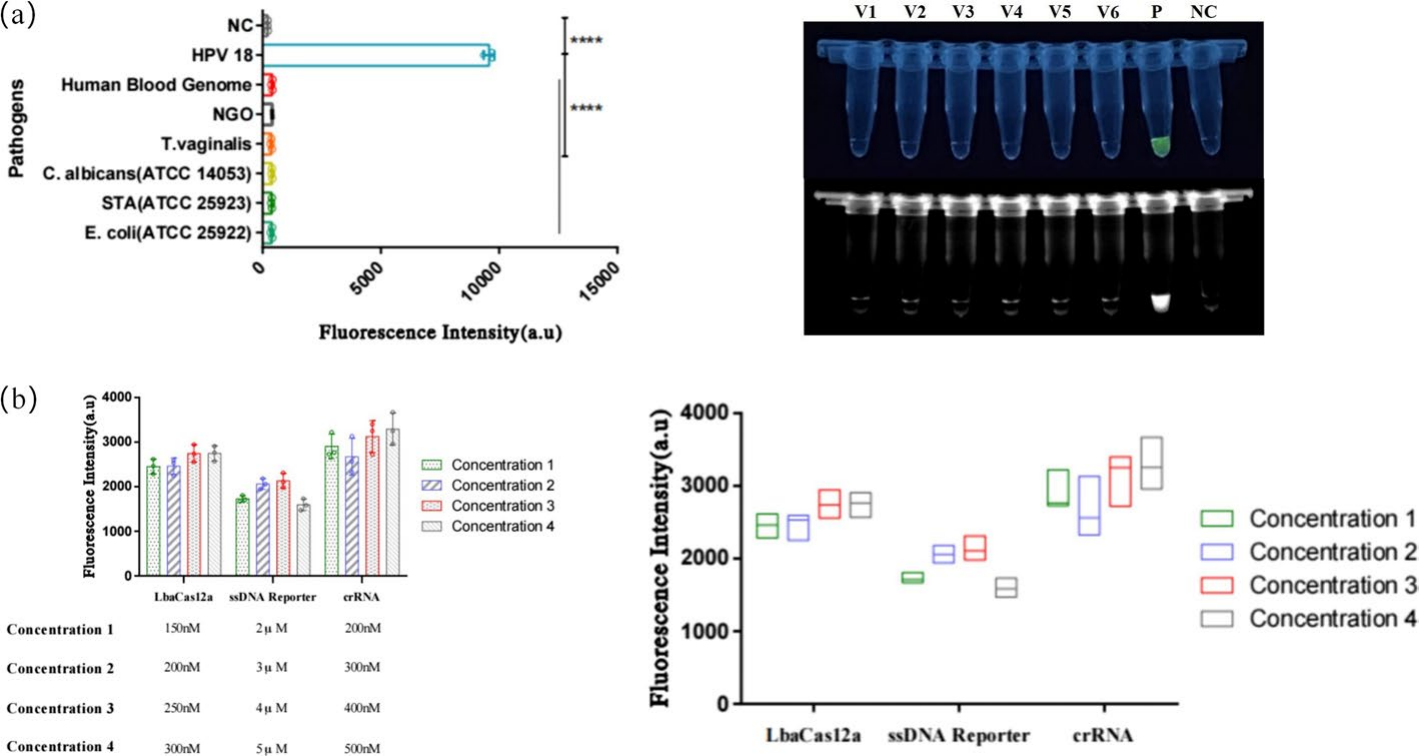
Specificity validation of the dual detection system H-MRC12a and optimization results for the components of the CRISPR system. **a** Specificity validation results for the H-MRC12a detection system against common pathogens in the female reproductive tract. After extracting nucleic acids from each pathogen, they were added to the reaction system, and fluorescence was observed immediately under UV light after a 35-min reaction. The results demonstrate no cross-reaction with the following five pathogens and the human blood genome: V1: *E. coli* (ATCC 25922), V2: *S. aureus* (ATCC 25923), V3: *C. albicans* (ATCC 14053), V4: *T. vaginalis*, V5: *N. gonorrhoeae* (NGO), V6: Human Blood Genome, *NC* negative control reaction, *P* positive control using HPV 18 as the DNA template. The asterisk indicates significant differences compared to P, as determined by two-tailed Student’s *t*-test (****P < 0.0001). **b** Comparison of fluorescence intensity for different concentrations of Lba Cas12a protein, ssDNA reporter, and crRNA in the CRISPR system. The optimal component concentrations were determined as follows: Lba Cas12a 250 nM, ssDNA reporter 4 μM, and crRNA 500 nM

Additionally, using the RPA product from an HPV 16 plasmid (10^4^ copies/μL) as a template, this study optimized the concentrations of various components within a single CRISPR reaction system. The components included LbaCas12a protein, ssDNA reporter, and crRNA. The results indicated that the optimal concentrations for the system’s maximum fluorescence endpoint were 250 nM for LbaCas12a, 4 μM for ssDNA reporter, and 500 nM for crRNA (Fig. 7b). The final composition of the single CRISPR reaction system was determined as follows: LbaCas12a (1 μM) 2.5 μL, ssDNA reporter solution (10 μM) 4 μL, NEB Buffer 2.1 1 μL, crRNA water solution (10 μM) 0.5 μL, RPA Template 1.5 μL. The remaining volume was made up with nuclease-free water to reach a total volume of 10 μL. The dual-system combined detection reaction was conducted at 37 °C. Utilizing a microfluidic chip and an integrated portable device, the on-site detection could be completed within 45 min.

### Investigation of specificity and sensitivity in the H-MRC12a assay

In this study, the specificity of the dual-combined reaction system was evaluated using clinical cervical fluid samples negative for HPV, blood cell samples (depleted of red blood cells), and the DNA from five common reproductive tract pathogens: Staphylococcus aureus (ATCC 25923), Escherichia coli (ATCC 25922), Candida albicans (ATCC 14053), Trichomonas vaginalis, and Neisseria gonorrhoeae (Fig. 7a). The primary objective was to validate whether the detection system would yield false-positive results for clinical negative samples and blood-containing cervical fluid samples, as well as to investigate potential cross-reactivity with common pathogens in gynecological conditions. The results indicated that there was no cross-reactivity with the mentioned pathogens and the human blood cell genome, demonstrating a high level of specificity for the detection system.

**Fig. 8:**
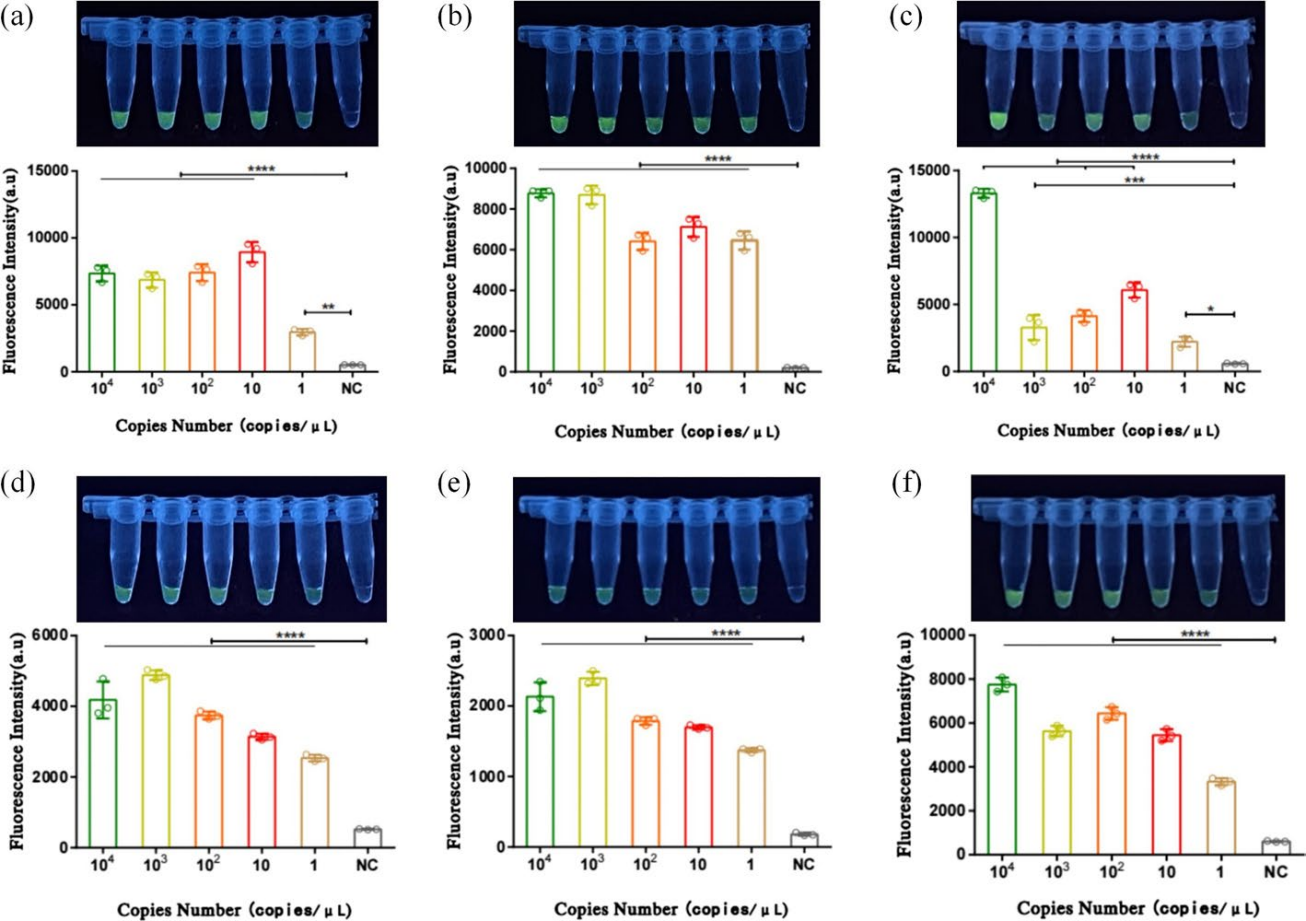
Sensitivity validation of the dual detection system H-MRC12a with a reaction time of 40 min (Multiple RPA 20 min + CRISPR 20 min). **a**–**f** Sensitivity validation results for different concentrations of plasmids for HPV types 16, 18, 31, 33, 35, and 45, respectively. The reaction results were observed immediately under UV light after completion. The asterisk indicates significant differences compared to the negative control, as determined by two-tailed Student’s *t*-test (*P < 0.1, ***P < 0.001, ****P < 0.0001). *ns* denotes no statistical significance. *NC* negative control reaction

In the assessment of sensitivity, this study utilized plasmids with concentrations ranging from 10^4^ copies/μL to 1 copy/μL for each of the six HR-HPV types as DNA templates. Following a 20-min multiplex RPA reaction, the CRISPR reaction was initiated immediately and allowed to proceed for an additional 20 min. Subsequently, fluorescence observation and analysis of the fluorescence curves were promptly conducted under ultraviolet light. The results indicated that the dual-combined detection system achieved a detection depth of 1 copy/μL for all six HPV types mentioned (Fig. 8).

### Examination of clinical sample

**Fig. 9:**
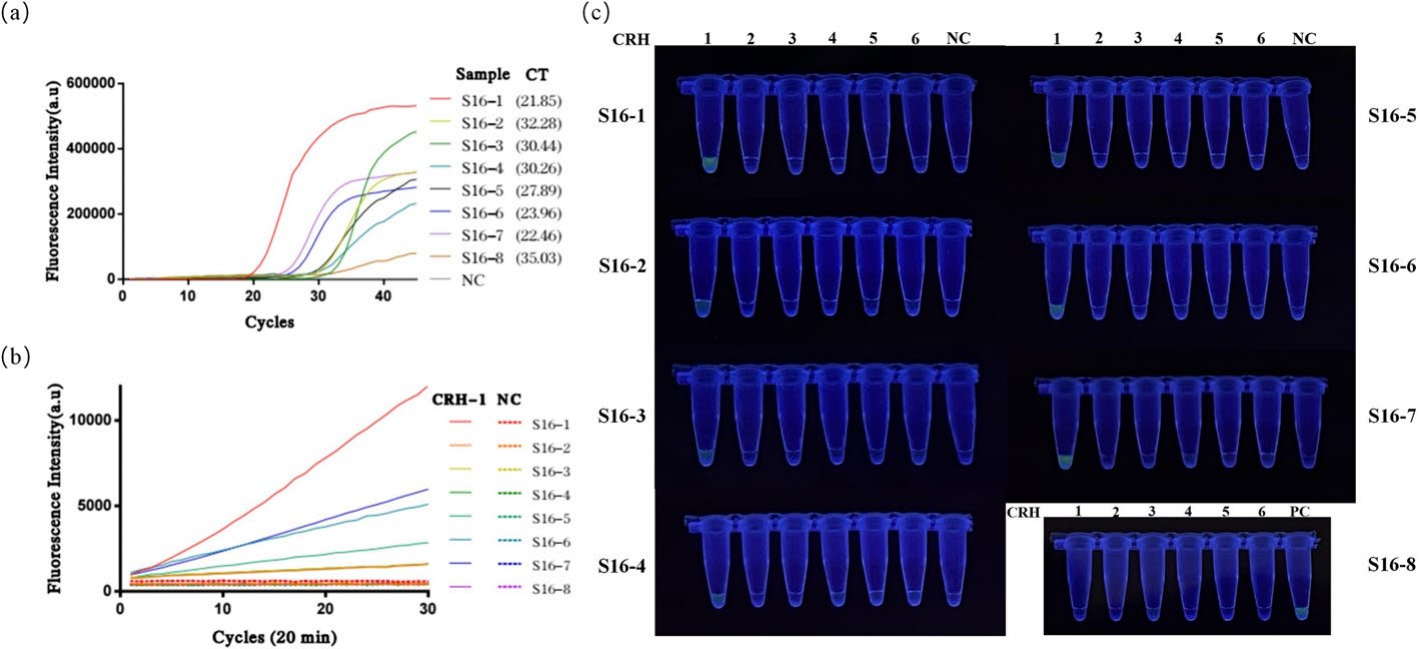
Comparison of experimental results between the H-MRC12a combined detection system and a commercially available QPCR genotyping kit for detecting 8 clinical HPV 16-positive samples. Clinical samples were heat-inactivated and subjected to nucleic acid extraction using the nanomagnetic bead method. The extracted nucleic acids were then incubated in the H-MRC12a combined detection system for 40 min (Multiple RPA incubation for 20 min, followed by CRISPR incubation for an additional 20 min) and immediately observed under 300 nm UV light. **a** Fluorescence curves and CT values obtained from the commercially available QPCR genotyping kit for the 8 clinical samples. **b** Real-time fluorescence signals (recorded using the Q160 LongGene portable QPCR instrument) of the H-MRC12a dual detection system for the clinical samples and negative control after 20 min of incubation in the CRISPR step. **c** Photographs taken under UV light of the 8 clinical samples incubated for 40 min in the H-MRC12a detection system. S16-1~S16-8: Identification numbers of the 8 HPV 16-positive clinical samples; CRH1~6: Tubes containing crRNA specific for HPV 16, 18, 31, 33, 35, and 45, respectively, for genotyping detection; NC: Negative control; PC: Positive control

**Fig. 10:**
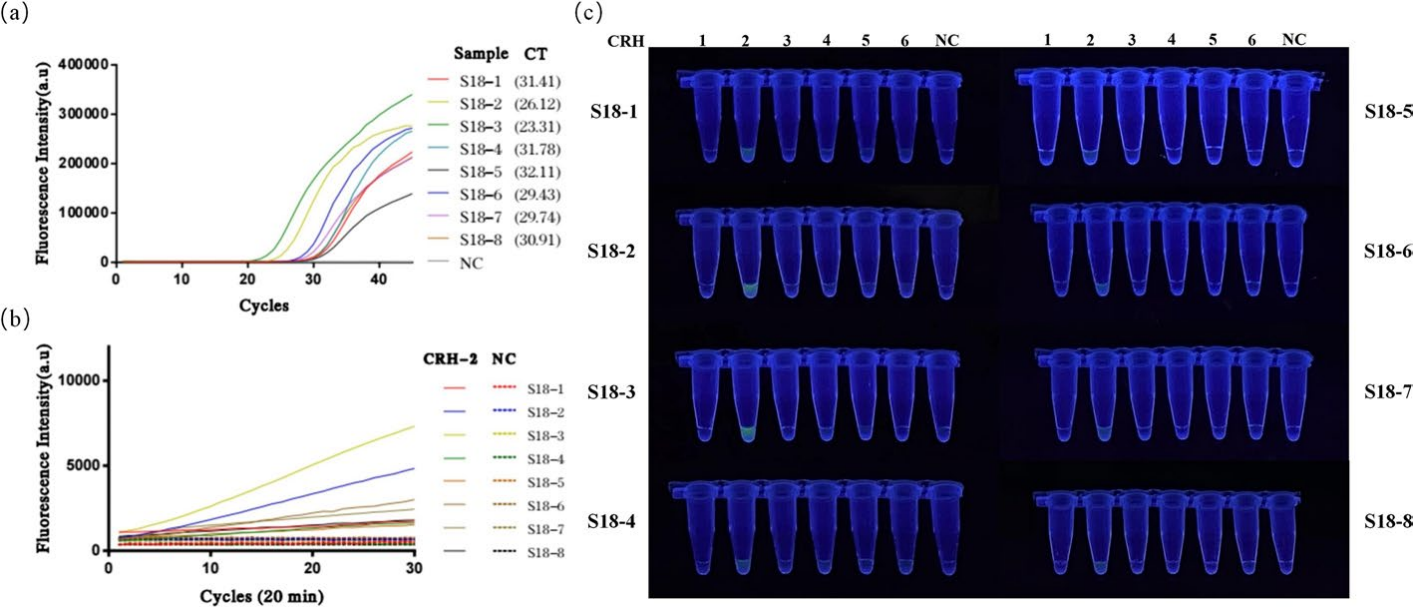
Comparison of experimental results between the H-MRC12a combined detection system and a commercially available QPCR genotyping kit for detecting 8 clinical HPV 18-positive samples. Clinical samples were heat-inactivated and subjected to nucleic acid extraction using the nanomagnetic bead method. The extracted nucleic acids were then incubated in the H-MRC12a combined detection system for 40 min (Multiple RPA incubation for 20 min, followed by CRISPR incubation for an additional 20 min) and immediately observed under 300 nm UV light. **a** Fluorescence curves and CT values obtained from the commercially available QPCR genotyping kit for the 8 clinical samples. **b** Real-time fluorescence signals (recorded using the Q160 LongGene portable QPCR instrument) of the H-MRC12a dual detection system for the clinical samples and negative control after 20 min of incubation in the CRISPR step. **c** Photographs taken under UV light of the 8 clinical samples incubated for 40 min in the H-MRC12a detection system. S18-1~S18-8: Identification numbers of the 8 HPV 18-positive clinical samples; CRH1~6: Tubes containing crRNA specific for HPV 16, 18, 31, 33, 35, and 45, respectively, for genotyping detection; NC: Negative control

**Fig. 11:**
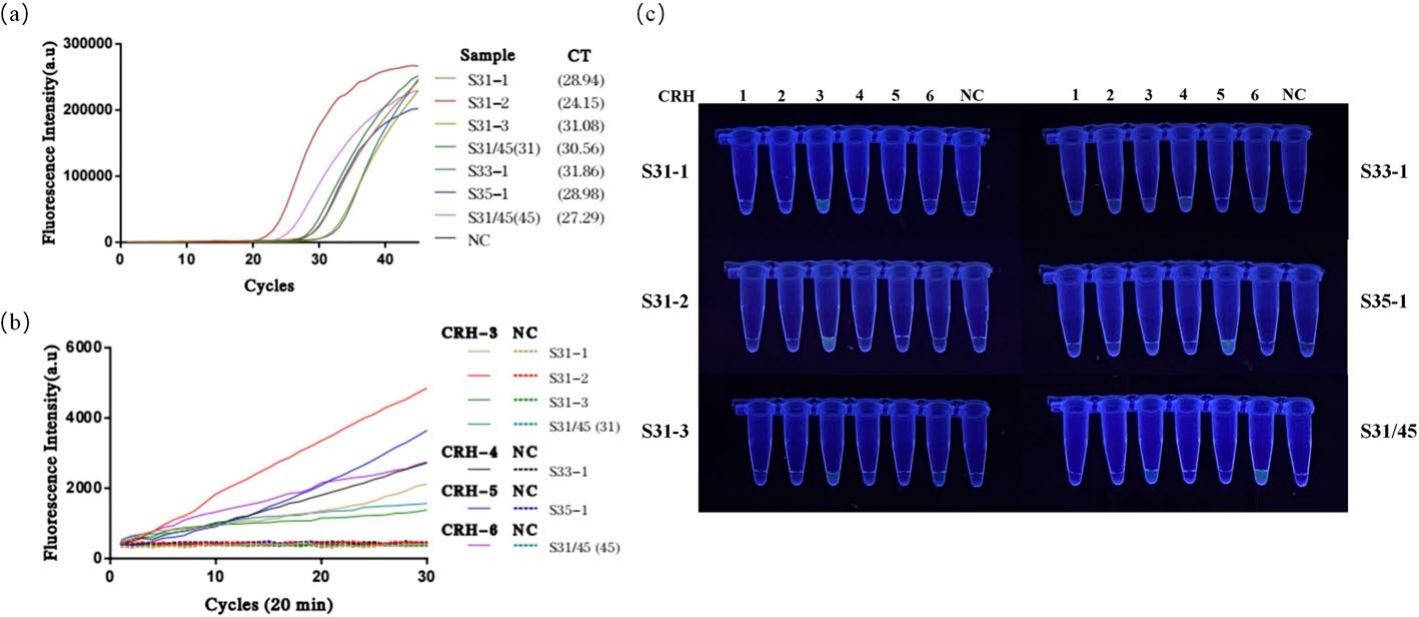
Comparison of experimental results between the H-MRC12a combined detection system and a commercially available QPCR genotyping kit for detecting clinical HPV 31, 33, 35, and 45-positive samples. Clinical samples were heat-inactivated and subjected to nucleic acid extraction using the nanomagnetic bead method. The extracted nucleic acids were then incubated in the H-MRC12a combined detection system for 40 min (Multiple RPA incubation for 20 min, followed by CRISPR incubation for an additional 20 min) and immediately observed under 300 nm UV light. **a** Fluorescence curves and CT values obtained from the commercially available QPCR genotyping kit for the clinical samples. **b** Real-time fluorescence signals (recorded using the Q160 LongGene portable QPCR instrument) of the H-MRC12a dual detection system for the clinical samples and negative control after 20 min of incubation in the CRISPR step. **c** Photographs taken under UV light of the clinical samples of each type incubated for 40 min in the H-MRC12a detection system. S31-1~S31-3: Identification numbers of 3 HPV 31-positive clinical samples; S33-1/S35-1: Identification numbers of HPV 33-positive and HPV 35-positive clinical samples; S31/45: Identification numbers of HPV 31/45 double-positive clinical samples; CRH1~6: Tubes containing crRNA specific for HPV 16, 18, 31, 33, 35, and 45, respectively, for genotyping detection; NC: Negative control

**Fig. 12:**
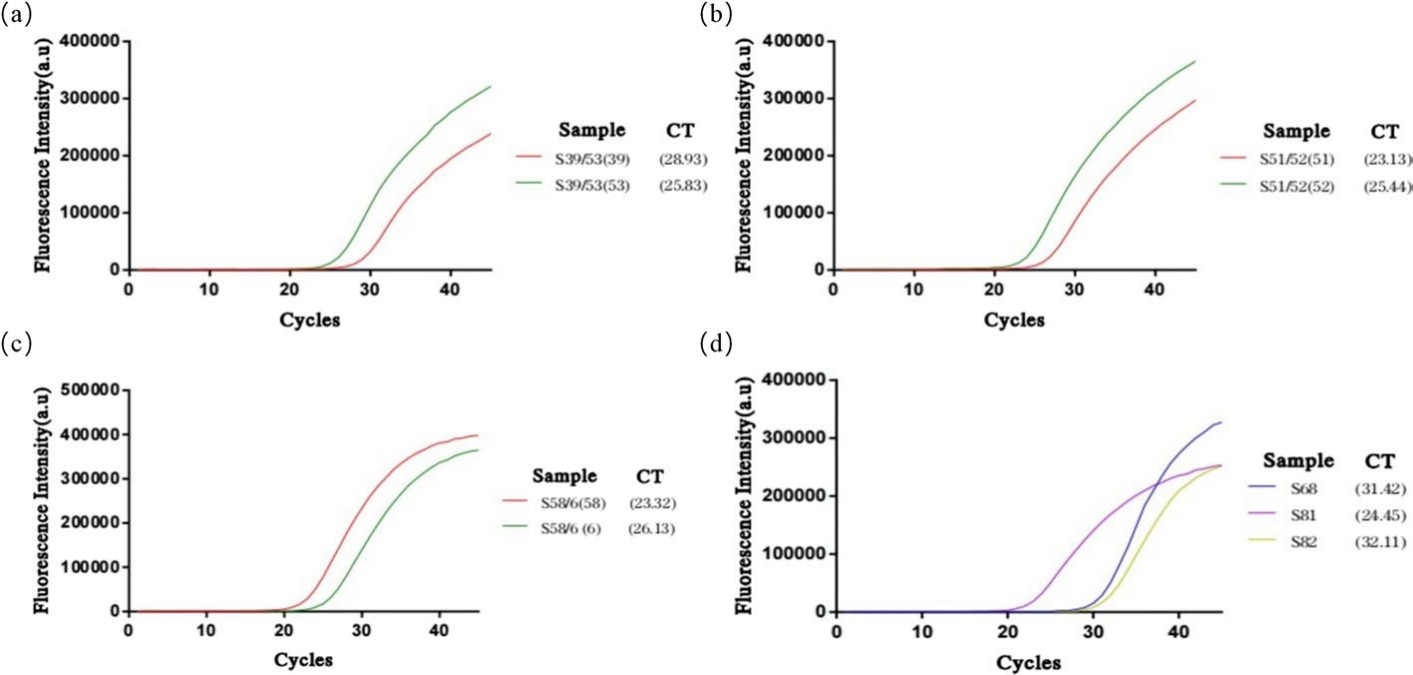
Fluorescence curves of clinical samples with different HPV subtypes detected using a commercially available QPCR genotyping kit. **a** Fluorescence curve of a clinical sample double-positive for HPV 39/53. **b** Fluorescence curve of a clinical sample double-positive for HPV 51/52. **c** Fluorescence curve of a clinical sample double-positive for HPV 58/6. **d** Fluorescence curves of three clinical samples positive for HPV 68, 81, and 82, respectively

This study validated the reliability of the H-MRC12a combined reaction system using 34 clinical HPV cervical fluid samples. The obtained results were compared with those from a commercially available QPCR assay kit, specifically the Shuoshi Biological HPV Nucleic Acid Typing Detection Kit. The QPCR kit typically has a detection limit around 10 copies/μL, with the exact limit varying from 34 to 36 based on CT values, depending on the specific HPV type. The validated samples included 8 HPV 16-positive, 8 HPV 18-positive, 4 HPV 31-positive, 1 HPV 33-positive, 2 HPV 35-positive, and 1 HPV 31/45 double-positive samples. For HPV 16-positive samples, the results of the H-MRC12a method indicated the capability to detect at least the positive samples with a CT value as low as 32.28 (Fig. 9). Similarly, for HPV 18-positive samples, the H-MRC12a method could detect at least the positive samples with a CT value as low as 32.11 (Fig. 10). Although the sample numbers for HPV 31, 33, 35, and 45 were limited, the H-MRC12a method successfully detected them, with the CT values and fluorescence curves for each sample shown in Fig. 11. Moreover, one HPV 31-positive and one HPV 35-positive sample were undetected by the QPCR method, while the H-MRC12a method showed fluorescence. The detection results were further confirmed through sequencing (Additional file 1: Figs. S1, S2). The possible reason for the discrepancy in detection results may be attributed to differences in the conservativeness of the amplification regions chosen by the two methods, and the occasional occurrence of rare mutations in HPV-related sequences at the primer positions in QPCR, leading to reduced sensitivity in amplification. In addition, this study validated the specificity of the H-MRC12a method using four different types of HPV-negative samples (containing blood, yellow turbidity, and clear cervical fluid) and six single or double-positive samples of other high/low-risk HPV types. The fluorescence curves and CT values for positive samples are depicted in Fig. 12. For the mentioned samples, the H-MRC12a method did not exhibit fluorescence, demonstrating high specificity. The QPCR amplification curves for all positive samples are provided in Additional file 1: Figs. S3–S6. In summary, the jointly developed detection system in this study showed a high level of consistency with the commercially available QPCR assay kit. Samples with discrepancies in the results between the two methods were confirmed as positive through sequencing, affirming the accuracy of the H-MRC12a combined detection system.

## Discussion

HPV exhibits a wide range of genotypes, with the full-length genomic sequences of all types approximately around 8000 bp, and high conservation in their sequences. In addition to the protein regions associated with the pathogenic mechanisms of E6 and E7, the L1 region is also referred to as a conserved sequence [20], with high conservation observed in the nucleotide sequences of these three regions [21, 22]. The E6 and E7 genes of HPV play crucial roles during the virus's invasion of host cells [23]. Both of these oncogenic sequences contain a CXXC zinc finger protein motif capable of forming zinc-coordinated complexes. Some members of this protein family, such as CXXC5, are implicated in the occurrence and progression of various malignancies, participating in biological processes such as cell differentiation, apoptosis, endothelial cell invasion, and energy metabolism [24]. The zinc finger protein motif is conserved in the E6 and E7 proteins of all HPV types, providing a reference for the primer design direction in this study. Therefore, the primer design and selection in this study prioritize regions that include the conserved zinc finger protein motif. Many commonly reported universal PCR primers (e.g., single primers GP5 + /GP6 + , degenerate primers MY09/11, etc.) are also designed in these regions[25]. While universal PCR primers targeting the L1 region (such as MY011) can effectively amplify a broad spectrum of HPV genotypes, there are challenges associated with their use. Firstly, these universal primers amplify HPV genotypes in an unpredictable manner, and some amplified regions are overly conserved, making it challenging for genotype studies. Secondly, modifying PCR primers into RPA primers presents several issues. RPA primers, which are nearly twice the length of PCR primers, impose higher demands on the sequence conservation at both ends of the universal primer extension direction. Additionally, the CRISPR/Cas12a system imposes strict requirements on the presence of Cas12a PAM sites within the amplified region. To fundamentally address the issue of multimer formation arising from sequence differences among multiple amplification primers, this study, inspired by the concept of “universal primers” [26], conducted a meticulous selection of amplicon regions and construction of primer pools. Building upon the highly conserved zinc finger protein motif, the study initially screened stable regions for HPV amplification. Subsequently, refined designs for primers targeting various HPV genotypes were developed based on the sequences flanking the selected regions. The chosen segments for amplification of each HPV genotype in this study were located at the same position in the entire genome and included multiple PAM sites. Among these PAM sites, more than 20 nucleotides extending in the 3’ direction from some sites exhibited high sequence conservation, while the 3’ direction extension of 20 nucleotides from other sites showed significant sequence differences. This provides the foundation for the specificity of crRNA selection in genotype detection and simultaneously offers the possibility of non-genotype detection. The stringent requirements also make the design and selection of RPA primers a central focus of this study. Currently, research in the field of HPV in POCT (Point-of-Care Testing) is predominantly limited to types 16/18. Researchers find it challenging to expand the detection of genotypes while maintaining the stability and sensitivity of a multiplex detection system, falling short of meeting the clinical demands for HPV detection. The RPA technology serves well to balance the demands of signal amplification and system stability. However, the issue of decreased sensitivity due to the mutual influence of primers in multiplex detection has been a persistent challenge in such amplification technologies. Researchers like Gong et al. [17] referenced multiple universal PCR primers to construct primer pools and crRNA pools capable of detecting various high-risk (HR) HPV types. However, this study did not fundamentally address the issue of multiple amplifications, as the excessive number and significant sequence differences among the primers led to a substantial decrease in sensitivity and stability. Moreover, the study did not integrate the two systems. In this research, we leveraged the conservation patterns in HPV sequences to attempt a novel solution to the challenge of multiple RPA primers, aiming to enhance the sensitivity of multiplex detection. By optimizing the CRISPR/Cas12a system and conducting extensive crRNA screening, we developed the H-MRC12a method, enabling high-specificity genotyping of six HR-HPV types. The design approach of “universal primers” resulted in extremely high sequence similarity among the primers, with most having only single-base differences. This effectively suppressed the formation of dimers between multiple primers, and adjusting the concentrations of individual primers significantly improved the sensitivity of multiplex RPA. In the optimization phase, to enhance the specificity of homologous recognition and the stability of chain exchange reactions in RPA, we introduced Betaine to stabilize the binding of the BSU polymerase with the template DNA chain. This aided the polymerase in navigating through complex base structures on the DNA chain [27], enhancing the stability of UvsX-ssDNA binding and promoting homologous recognition processes. Consequently, this optimization further improved the stability and sensitivity of the preceding RPA reaction, even raising the detection copy number of HPV type 45 by an order of magnitude.

Various Cas proteins within the CRISPR system find applications in the detection field, such as DETECTR [28], SHERLOCK [29], SHERLOCK_v2_ [30], HOLMES [31] and others. Research in the molecular biology discipline related to HPV treatment is diverse and well-established [32], while studies utilizing the CRISPR system for HPV detection are still in their infancy. As a composite system that combines biological sensing and signal amplification functions, CRISPR/Cas extensions in both aspects are relatively limited. When applied in the field of Point-of-Care Testing (POCT), the biological sensing aspect requires suitable downstream biosensing methods such as fluorescent probes [33–35], electrochemistry [36, 37], spectroscopy [38], and other relevant technologies to achieve molecular signal conversion and thereby complete the presentation of POCT results. To further enhance the depth of detection, the CRISPR system needs to be coupled with technologies that enable signal amplification as a preliminary step, such as LAMP [39], RPA [40, 41], SERS [42] or other techniques. Regardless of the technology employed, achieving true Point-of-Care (POC) genotyping requires an integrated medium that harmonizes various techniques. Microfluidic chip technology [43, 44] serves as one of the numerous options for such integration. Other directions of exploration include the use of lateral flow-based paper devices [45, 46] or microfluidic dual-droplet devices [47] as carriers. In recent years, research on POC testing for human papillomavirus (HPV) has predominantly focused on innovation within the CRISPR system and its subsequent biosensing steps. Han et al. [48] designed a system where Blocker DNA binds to probe ssDNA, regulating fluorescence signals from different probes through reverse transcription-generated ssRNA. This approach enables genotyping of HPV within a unified system. Additionally, Chen et al. [49] identified a novel specific Cas12a PAM site: TTNA. Moreover, the sensitivity issues challenging multiplex RPA have prompted many researchers to explore amplification-free signal amplification techniques to assist the CRISPR system in achieving deep detection. Xue et al. [50] introduced an amplification-free HPV detection technology that combines dispersed droplets with CRISPR. Yu et al. [37] employed DNA tetrahedral nanostructures (TDNs) in an electrochemiluminescence biosensing technique to assist CRISPR in achieving amplification-free signal amplification and secondary sensing. However, these studies have primarily focused on HPV types 16/18 or solely used HPV as a validation template, falling short in meeting broader detection needs. Considering the multitude of HPV types, achieving Point-of-Care (POC) genotyping for high-risk (HR) HPV is a crucial prerequisite for HPV risk stratification management. Given the viral characteristics of HPV and the reaction temperature of RPA, this study selected Cas12a, capable of active function at 37 ℃ and direct recognition of DNA target sequences, as the core functional protein. By designing and screening specific crRNAs, this research conducted genotyping tests for six HR-HPV types. Due to the significant impact of the stability of crRNA-target binding on the cleavage activity of Cas proteins [51], this study introduced a specially designed “blocking” sequence at the gRNA’s end to prevent potential off-target effects after crRNA binding to the target sequence and to enhance its base discrimination capability. The blocking structure, by adopting a highly distorted conformation, tightly binds to the nucleic acid-binding domain of the Cas protein, stabilizing the Cas protein conformation [52] and improving the specificity of the CRISPR system. Research indicates that the introduction of the blocking structure in crRNA enhances the base discrimination capability of the CRISPR system by at least threefold. Furthermore, variations in the blocking structure sequence also affect the base recognition capability of crRNA [53]. In the CRISPR reaction system, after gRNA pairs with the target, it activates the nucleolytic domain of Cas12a [54]. This activation prompts Cas protein to indiscriminately cleave the FAM ssDNA probe in the system and emit a fluorescent signal. Under 300 nm wavelength UV light exposure, visual determination of effective genotyping results for various HPV types can be achieved. Additionally, this study observed that an excessive number of crRNA types in a single system significantly impairs system stability, leading to severe false-positive results. Thanks to the conservativeness of the PAM site sequences in the selected amplicon of this study, the method can also adopt a "universal" approach for crRNA design to drastically simplify the crRNA pool, thereby constructing a highly stable non-genotyping detection method.

In summary, this study successfully constructed a highly sensitive multiplex RPA amplification system targeting multiple high-risk (HR) HPV types by leveraging the regularities in the HPV genomic sequences. Combined with the optimized CRISPR/Cas12a system, the developed system can accomplish genotyping detection for six HR-HPV types within 40 min at 37 °C, allowing for on-site visual interpretation. The detection depth for each type reaches 1 copy/μL. The developed method has enormous potential for further improvement in detection throughput or flexible conversion into non-genotyping detection based on the provided primer design strategy. Therefore, this research contributes to the development of Point-of-Care (POC) high-risk HPV genotyping detection, promoting the prevalence of HPV testing. Moreover, many concepts developed during the construction of the H-MRC12a combined detection system can serve as inspiration and guidance for the POC research of other pathogens.

### Supplementary Information

**Additional file 1: Table S1.** Sequences of plasmids. **Table S2.** Sequences of primers. **Table S3.** Coverage of HPV 16/18/31/33/35/45 crRNAs used for the H-MRC12a. **Figure S1.** H-MRC12a method detection results and sequencing alignment of the RPA amplification product for an HPV 31-positive clinical sample not detected by the QPCR method. **a** The sample, after heat inactivation, underwent nucleic acid extraction using the nanomagnetic bead method. The extracted nucleic acids were incubated in the H-MRC12a combined detection system for 40 mins (Multiple RPA incubation for 20 mins, followed by CRISPR incubation for an additional 20 mins) and immediately observed under 300 nm UV light. **b** Real-time fluorescence signals (recorded using the Q160 LongGene portable QPCR instrument) of the clinical sample and negative control after 20 mins of incubation in the CRISPR step. **c** Bar chart of the fluorescence endpoint values for the clinical sample and negative control after 20 mins of incubation in the CRISPR step. **d** Sequencing results of the RPA amplification product for the clinical sample. **e** Alignment of the base sequence obtained from sequencing of the clinical sample with the NCBI database. The comprehensive results indicate a high similarity between the RPA amplification product sequence of the sample and various HPV 31 sequences in the NCBI database. **Figure S2.** H-MRC12a method detection results and sequencing alignment of the RPA amplification product for an HPV 35-positive clinical sample not detected by the QPCR method. **a** The sample, after heat inactivation, underwent nucleic acid extraction using the nanomagnetic bead method. The extracted nucleic acids were incubated in the H-MRC12a combined detection system for 40 mins (Multiple RPA incubation for 20 mins, followed by CRISPR incubation for an additional 20 mins) and immediately observed under 300 nm UV light. **b** Real-time fluorescence signals (recorded using the Q160 LongGene portable QPCR instrument) of the clinical sample and negative control after 20 mins of incubation in the CRISPR step. **c** Bar chart of the fluorescence endpoint values for the clinical sample and negative control after 20 mins of incubation in the CRISPR step. **d** Sequencing results of the RPA amplification product for the clinical sample. **e** Alignment of the base sequence obtained from sequencing of the clinical sample with the NCBI database. The comprehensive results indicate a high similarity between the RPA amplification product sequence of the sample and various HPV 35 sequences in the NCBI database. **Figure S3.** QPCR amplification curves for eight clinical samples positive for HPV 16, labeled as S16-1 to S16-8. **Figure S4.** QPCR amplification curves for eight clinical samples positive for HPV 18, labeled as S18-1 to S18-8. **Figure S5.** QPCR amplification curves for three clinical samples positive for HPV 31, labeled as S31-1 to S31-3, and for HPV 33, 35, and 31/45 double-positive clinical samples labeled as S33-1, S35-1, and S31/45, respectively. **Figure S6.** QPCR amplification curves for clinical samples positive for various additional high/low-risk HPV types, detected by the H-MRC12a method. S39/53: HPV 31/53 double-positive sample. S51/52: HPV 51/52 double-positive sample. S58/6: HPV 58/6 double-positive sample. S68, S81, S82: Samples positive for HPV 68, 81, and 82, respectively.

## Data Availability

All data generated or analysed during this study are included in this published article and its Additional information files.

## Abbreviations

HR-HPV: High-risk human papillomavirus
POC: Point of care
POCT: Point of care testing
RPA: Recombinase polymerase amplification
CRISPR: Clustered regularly interspaced short palindromic repeats
CRISPR/Cas12a: Cas12a protein of clustered regularly interspaced short palindromic repeats
H-MRC12a: Human papillomavirus—multiple recombinase polymerase amplification—clustered regularly interspaced short palindromic repeats/Cas12a
PCR: Polymerase chain reaction
QPCR: Quantitative real-time polymerase chain reaction
LAMP: Loop mediated isothermal amplification
RCA: Rolling circle amplification
RAA: Recombinase-aidamplification
crRNA: CRISPR RNA
gRNA: Graduate RNA
ssDNA: Single-stranded DNA
ssRNA: Single-stranded RNA
PAM: Protospacer adjacent motif
NCBI: National center for biotechnology information
FAM: Carboxyfluoresceinr
CT value: Cycle threshold value
DETECTR: DNA endonuclease-targeted CRISPR trans reporter
SHERLOCK: Specific high-sensitivity enzymatic reporter unlocking
SHERLOCK_v2_: Specific high sensitivity enzymatic reporter unlocking version 2
HOLMES: One-hour low-cost multipurpose highly efficient system
SERS: Surfaced enhanced Raman spectroscopy
TDNs: DNA tetrahedral nanostructures
