# Correction: A multiplex RPA-CRISPR/Cas12a-based POCT technique and its application in human papillomavirus (HPV) typing assay

**DOI:** 10.1186/s11658-024-00567-9

**Published:** 2024-04-09

**Authors:** Yan Liu, Zhujun Chao, Wei Ding, Tanfeng Fang, Xinxian Gu, Man Xue, Wei Wang, Rong Han, Wanping Sun

**Affiliations:** 1 Laboratory of Molecular Diagnostics, College of Pharmaceutical Sciences, Soochow University, Suzhou, 215000 Jiangsu People’s Republic of China; 2 Soochow University, Suzhou Medical College of Soochow University, Suzhou, 215000 Jiangsu People’s Republic of China; 3 Dushu Lake Hospital, Affiliated to Soochow University, Dushu Lake Hospital Affiliated to Soochow University, Suzhou, 215004 Jiangsu People’s Republic of China; 4 Biological Products and Biochemical Drugs, Suzhou Institute for Food and Drug Control, Suzhou, 215101 Jiangsu People’s Republic of China

**Correction: Cellular & Molecular Biology Letters (2024) 29:34** 10.1186/s11658-024-00548-y

Following publication of the original article [1], we have been informed that the Supplementary Information is incomplete.

The original article has been corrected.
