# Supplementary material for: A multiplex RPA-CRISPR/Cas12a-based POCT technique and its application in human papillomavirus (HPV) typing assay
Source: Cell Mol Biol Lett. 2024 Mar 8;29:34. doi: 10.1186/s11658-024-00548-y (PMC10921630; doi:10.1186/s11658-024-00548-y)

Table S1. Sequences of plasmids

| **Postive stangard** | **Source** | **Location** | **Sequences(5’~3’)** |
| --- | --- | --- | --- |
| HPV 16 | KX947282.1 | 6506—7059 | ATTATTTTCCTACACCTAGTGGTTCTATGGTTACCTCTGATGCCCAAATATTCAATAAACCTTATTGGTTACAACGAGCACAGGGCCACAATAATGGCATTTGTTGGGGTAACCAACTATTTGTTACTGTTGTTGATACTACACGCAGTACAAATATGTCATTATGTGCTGCCATATCTACTTCAGAAACTACATATAAAAATACTAACTTTAAGGAGTACCTACGACATGGGGAGGAATATGATTTACAGTTTATTTTTCAACTGTGCAAAATAACCTTAACTGCAGACGTTATGACATACATACATTCTATGAATTCCACTATTTTGGAGGACTGGAATTTTGGTCTACAACCTCCCCCAGGAGGCACACTAGAAGATACTTATAGGTTTGTAACATCCCAGGCAATTGCTTGTCAAAAACATACACCTCCAGCACCTAAAGAAGATCCCCTTAAAAAATACACTTTTTGGGAAGTAAATTTAAAGGAAAAGTTTTCTGCAGACCTAGATCAGTTTCCTTTAGGACGCAAATTTTTACTACAAGCAGGATT |
| HPV 18 | GQ180792.1 | 6480—7039 | TGTATTCTCCCTCTCCAAGTGGCTCTATTGTTACCTCTGACTCCCAGTTGTTTAATAAACCATATTGGTTACATAAGGCACAGGGTCATAACAATGGTGTTTGCTGGCATAATCAATTATTTGTTACTGTGGTAGATACCACTCGCAGTACCAATTTAACAATATGTGCTTCTACACAGTCTCCTGTACCTGGGCAATATGATGCTACCAAATTTAAGCAGTATAGCAGACATGTTGAGGAATATGATTTGCAGTTTATTTTTCAGTTGTGTACTATTACTTTAACTGCAGATGTTATGTCCTATATTCATAGTATGAATAGCAGTATTTTAGAGGATTGGAACTTTGGTGTTCCCCCCCCGCCAACTACTAGTTTGGTGGATACATATCGTTTTGTACAATCTGTTGCTATTACCTGTCAAAAGGATGCTGCACCGGCTGAAAATAAGGATCCCTATGATAAGTTAAAGTTTTGGAATGTGGATTTAAAGGAAAAGTTTTCTTTAGACTTAGATCAATATCCCCTTGGACGTAAATTTTTGGTTCAGGCTGGATTGCG |
| HPV 31 | LR862053.1 | 6080~6950 | GATTGTCCTCCATTAGAATTAAAAAATTCAGTTATACAAGATGGGGATATGGTTGATACAGGCTTTGGAGCTATGGATTTTACTGCTTTACAAGACACTAAAAGTAATGTTCCTTTGGACATTTGTAATTCTATTTGTAAATATCCAGATTATCTTAAAATGGTTGCTGAGCCATATGGCGATACATTATTTTTTTATTTACGTAGGGAACAAATGTTTGTAAGACATTTTTTTAATAGATCAGGCGCGGTTGGTGAATCGGTTCCTAATGACTTATATATTAAAGGCTCCGGTTCAACAGCTACTTTAGCTAACAGTACATACTTTCCTACACCTAGCGGCTCCATGGTTACTTCAGATGCACAAATTTTTAATAAACCATATTGGATGCAACGTGCTCAGGGACACAATAATGGTATTTGTTGGGGCAATCAGTTATTTGTTACTGTGGTAGATACCACACGTAGTACCAATATGTCTGTGTGTGCTGCAATTGCAAACAGTGATACTACATTTAAAAGTAGTAATTTTAAAGAGTATTTAAGACATGGTGAGGAATTTGATTTACAATTTATATTTCAGTTATGCAAAATAACATTATCTGCAGACATAATGACATATATTCACAGTATGAATCCTGCTATTTTGGAAGATTGGAATTTTGGATTGACCACACCTCCCTCAGGTTCTTTAGAGGATACCTATAGGTTTGTAACCTCACAGGCCATTACATGTCAAAAAACTGCCCCCCAAAAGCCCAAGGAAGATCCATTTAAAGATTATGTATTTTGGGAGGTTAATTTAAAAGAAAAGTTTTCTGCAGATTTAGATCAGTTTCCACTGGGTCGCAAATTTTTATTACAGGCAGGA |

| **Postive stangard** | **Source** | **Location** | **Sequences(5’~3’)** |
| --- | --- | --- | --- |
| HPV 33 | LR862077.1 | 6199~6948 | GACACAGGATTTGGTTGCATGGATTTTAAAACATTGCAGGCTAATAAAAGTGATGTTCCTATTGATATTTGTGGCAGTACATGCAAATATCCAGATTATTTAAAAATGACTAGTGAGCCTTATGGTGATAGTTTATTTTTCTTTCTTCGACGTGAACAAATGTTTGTAAGACACTTTTTTAATAGGGCTGGTACATTAGGAGAGGCTGTTCCCGATGACCTGTACATTAAAGGTTCAGGAACTACTGCCTCTATTCAAAGCAGTGCTTTTTTTCCCACTCCTAGTGGATCAATGGTTACTTCCGAATCTCAGTTATTTAATAAGCCATATTGGCTACAACGTGCACAAGGTCATAATAATGGTATTTGTTGGGGCAATCAGGTATTTGTTACTGTGGTAGATACCACTCGCAGTACTAATATGACTTTATGCACACAAGTAACTAGTGACAGTACATATAAAAATGAAAATTTTAAAGAATATATAAGACATGTTGAAGAATATGATCTACAGTTTGTTTTTCAACTATGCAAAGTTACCTTAACTGCAGAAGTTATGACATATATTCATGCTATGAATCCAGATATTTTAGAAGATTGGCAATTTGGTTTAACACCTCCTCCATCTGCTAGTTTACAGGATACTTATAGGTTTGTTACCTCTCAGGCTATTACGTGTCAAAAAACAGTACCTCCAAAGGAAAAGGAAGACCCCTTAGGTAAATATACATTTTGGGAAGTGGATTTAAA |
| HPV 35 | LR862022.1 | 6202-6953 | AGACACAGGATTTGGTGCAATGGATTTTACTACATTACAAGCTAATAAAAGTGATGTTCCCCTAGATATATGCAGTTCCATTTGCAAATATCCTGATTATCTAAAAATGGTTTCTGAGCCATATGGAGATATGTTATTTTTTTATTTACGTAGGGAGCAAATGTTTGTTAGACATTTATTTAATAGGGCTGGAACTGTAGGTGAAACAGTACCTGCAGACCTATATATTAAGGGTACCACTGGCACATTGCCTAGTACTAGTTATTTTCCTACTCCTAGTGGCTCTATGGTAACCTCCGATGCACAAATATTTAATAAACCATATTGGTTGCAACGTGCACAAGGCCATAATAATGGTATTTGTTGGAGTAACCAATTGTTTGTTACTGTAGTTGATACAACCCGTAGTACAAATATGTCTGTGTGTTCTGCTGTGTCTTCTAGTGACAGTACATATAAAAATGACAATTTTAAGGAATATTTAAGGCATGGTGAAGAATATGATTTACAGTTTATTTTTCAGTTATGTAAAATAACACTAACAGCAGATGTTATGACATATATTCATAGTATGAACCCGTCCATTTTAGAGGATTGGAATTTTGGCCTTACACCACCGCCTTCTGGTACCTTAGAGGACACATATCGCTATGTAACATCACAGGCTGTAACTTGTCAAAAACCCAGTGCACCAAAACCTAAAGATGATCCATTAAAAAATTATACTTTTTGGGAGGTTGATTTAAAGGAA |
| HPV 45 | LR862061.1 | 6212-6966 | GATACAGGTTATGGGGCAATGGATTTTAGTACATTGCAGGATACAAAGTGCGAGGTTCCATTAGACATTTGTCAATCCATCTGTAAATATCCAGATTATTTGCAAATGTCTGCTGATCCCTATGGGGATTCTATGTTTTTTTGCCTACGCCGTGAACAATTGTTTGCAAGACATTTTTGGAATAGGGCAGGTGTTATGGGTGACACGGTACCTACAGACCTATATATTAAAGGCACAAGCGCTAATATGCGTGAAACCCCTGGCAGTTGTGTGTATTCCCCTTCTCCCAGTGGCTCTATTATTACATCTGATTCTCAATTATTTAATAAGCCATATTGGTTACATAAGGCCCAGGGCCATAACAATGGTATTTGTTGGCATAATCAGTTGTTTGTTACTGTAGTGGACACTACCCGCAGTACTAATTTAACATTATGTGCCTCTACACAAAATCCTGTGCCAGGTACATATGATCCTACTAAGTTTAAGCAATATAGTAGACATGTGGAGGAATATGATTTACAGTTTATTTTTCAGTTGTGCACTATTACTTTAACTGCAGAGGTTATGTCATATATTCATAGTATGAATAGTAGTATATTGGAAAATTGGAATTTTGGTGTCCCTCCACCACCTACTACAAGTTTAGTGGATACATATCGTTTTGTGCAATCAGTTGCTGTTACCTGTCAAAAGGATACTACACCTCCAGAAAAGCAGGATCCATATGATAAATTAAAGTTTTGGACTGTT |

Table S2. Sequences of Primers

| **Forward** | **Primer** | **Sequences(5’~3’)** | **Length,nt** |
| --- | --- | --- | --- |
|  | HF1 | GAATTTAGATGGTATAGATATAGTGTGTATGGA | 33 |
|  | HF2 | GAATATAGACGTTATACAGACTCTGTGTATGGA | 33 |
|  | HF3 | TTTGTGACTGCGGTAGATACCACTCGCAGTAC | 32 |
|  | HF4 | TTTGTCACTGTCGTTGATACCACACGCAGTAC | 32 |
|  | HF5 | ACTAGACCATATTGGTTACAACGTGCACAGGG | 32 |
|  | HF6 | GATGCACAGATATTTAATAAACCTTATTGG | 30 |
|  | HF7 | GCAACCAGTTATTTGTTACTGTGGTTGATAC | 31 |
|  | | | |
| **Reverse** | HR1 | CCTGTCCACCGTCCACCTATGTTATGGAATCGT | 33 |
|  | HR2 | CCTGTCTAGCGTCCAGCTATGTTGTGGAATCGT | 33 |
|  | HR3 | ATTAGTACTGCGTGTGGTATCAACCACAGTAAC | 33 |
|  | HR4 | TAAACTGTAAATCATATTCCTCACCATGTC | 30 |
|  | HR5 | CATACTATGAATGTATGTCATAACGTCTGC | 30 |
|  | HR6 | CATAGCATGAATATATGTCATAACGTCTGCAG | 32 |
|  | HR7 | GTATGTATGTAGGACATAACGTCTGCAGTTA | 32 |
|  | HR8 | TCTGCAGTTAAGTTAAGGTAACTTTGCACAGT | 32 |
| The mentioned primers were employed for the stability screening of the amplification region using plasmids with HPV16 and 18 types as templates. Plasmids for both HPV types were constructed based on the respective NCBI HPV full genome sequences (Reference sequence number for HPV16: KX947282.1; Reference sequence number for HPV18: GQ180792.1). | | | |

Table S3. Coverage of HPV 16/18/31/33/35/45 crRNAs used for the H-MRC12a.

| **crRNA name** | **rename** | **Target Sequences** | **Genotype** |
| --- | --- | --- | --- |
| crR16-1 | _ | TTTG TTACTGTTGTTGATACTAC | HPV 16 |
| crR16-2 | _ | TTTG TTGGGGTAACCAACTATTT |
| crR16-3 | _ | TTTG TACTGCGTGTAGTATCAACAACAG |
| crR16-4 | CRH-1 | TTTC TGAAGTAGATATGGCAGCACATAA |
| crR16-5 | _ | TTTA TATGTAGTTTCTGAAGTAGATATG |
| crR18-1 | _ | TTTG TTACTGTGGTAGATACCAC | HPV 18 |
| crR18-2 | _ | TTTG CTGGCATAATCAATTATTT |
| crR18-3 | CRH-2 | TTTA ACAATATGTGCTTCTACACAGTCT |
| crR18-4 | _ | TTTG GTAGCATCATATTGCCCAGGTACA |
| crR18-5 | _ | TTTA AGCAGTATAGCAGACATGTTGAGG |
| crR31-1 | _ | TTTG TTACTGTGGTAGATACCAC | HPV 31 |
| crR31-2 | _ | TTTG TTGGGGCAATCAGTTATTT |
| crR31-3 | _ | TTTG CAATTGCAGCACACACAGACAT |
| crR31-4 | CRH-3 | TTTA AAAGTAGTAATTTTAAAGAGTATT |
| crR31-5 | _ | TTTA AATGTAGTATCACTGTTTGCAATT |
| crR33-1 | _ | TTTG TTACTGTGGTAGATACCAC | HPV 33 |
| crR33-2 | _ | TTTG TTGGGGCAATCAGGTATTT |
| crR33-3 | CRH-4 | TTTA TGCACACAAGTAACTAGTGACA |
| crR33-4 | _ | TTTA TATGTACTGTCACTAGTTACTTGT |
| crR33-5 | _ | TTTA AAGAATATATAAGACATGTTGAA |
| crR35-1 | CRH-5 | TTTG TTACTGTAGTTGATACAAC | HPV 35 |
| crR35-2 | _ | TTTG TTGGAGTAACCAATTGTTTGTTAC |
| crR35-3 | _ | TTTG TTACTGTAGTTGATACAACCCGTA |
| crR35-4 | _ | TTTG TACTACGGGTTGTATCAACTACAG |
| CrR45-1 | CRH-6 | TTTG TTACTGTAGTGGACACTAC | HPV 45 |

Figure S1. H-MRC12a method detection results and sequencing alignment of the RPA amplification product for an HPV 31-positive clinical sample not detected by the QPCR method. **(a)** The sample, after heat inactivation, underwent nucleic acid extraction using the nanomagnetic bead method. The extracted nucleic acids were incubated in the H-MRC12a combined detection system for 40 minutes (Multiple RPA incubation for 20 minutes, followed by CRISPR incubation for an additional 20 minutes) and immediately observed under 300nm UV light. **(b)** Real-time fluorescence signals (recorded using the Q160 LongGene portable QPCR instrument) of the clinical sample and negative control after 20 minutes of incubation in the CRISPR step. **(c)** Bar chart of the fluorescence endpoint values for the clinical sample and negative control after 20 minutes of incubation in the CRISPR step. **(d)** Sequencing results of the RPA amplification product for the clinical sample. **(e)** Alignment of the base sequence obtained from sequencing of the clinical sample with the NCBI database. The comprehensive results indicate a high similarity between the RPA amplification product sequence of the sample and various HPV 31 sequences in the NCBI database.


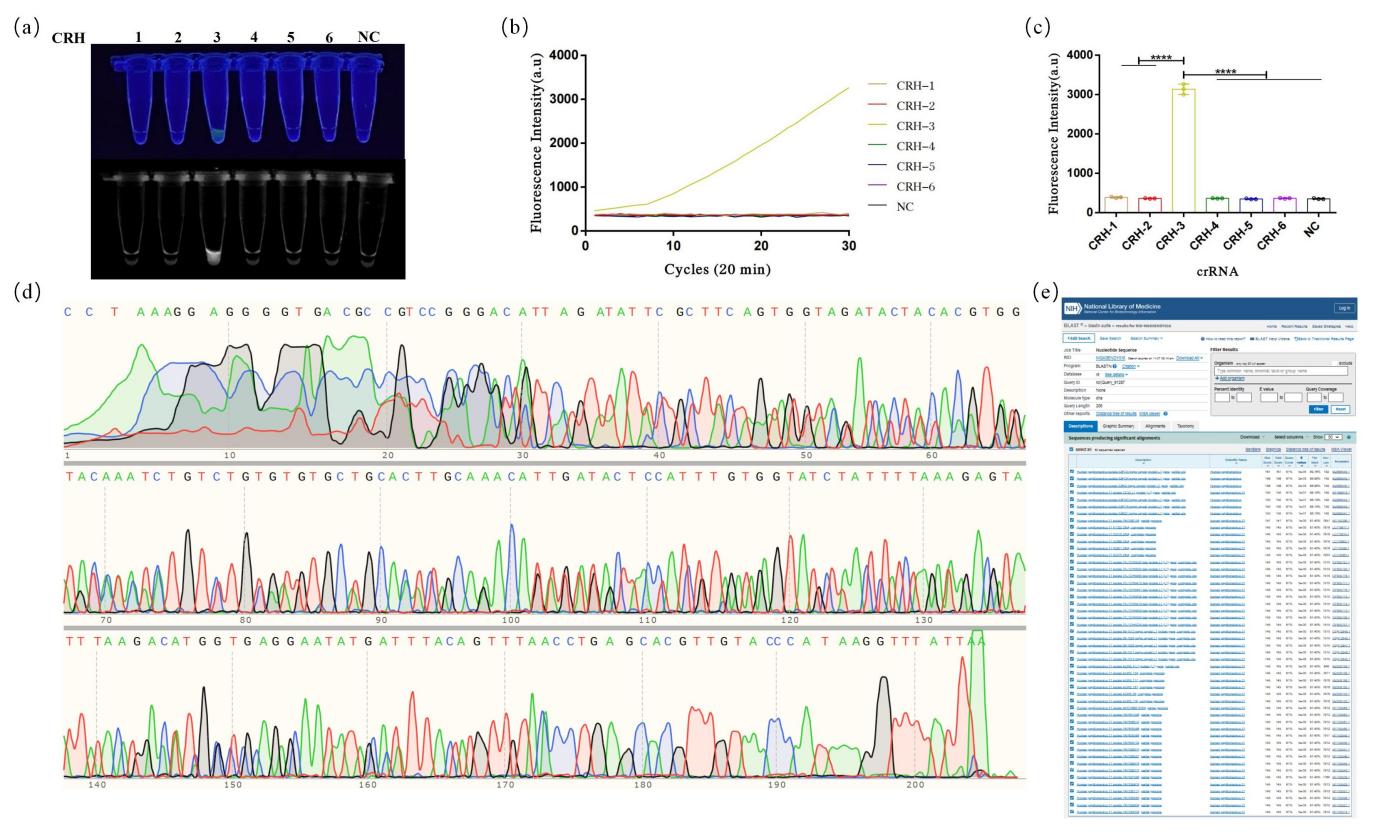


Figure S2. H-MRC12a method detection results and sequencing alignment of the RPA amplification product for an HPV 35-positive clinical sample not detected by the QPCR method. **(a)** The sample, after heat inactivation, underwent nucleic acid extraction using the nanomagnetic bead method. The extracted nucleic acids were incubated in the H-MRC12a combined detection system for 40 minutes (Multiple RPA incubation for 20 minutes, followed by CRISPR incubation for an additional 20 minutes) and immediately observed under 300nm UV light. **(b)** Real-time fluorescence signals (recorded using the Q160 LongGene portable QPCR instrument) of the clinical sample and negative control after 20 minutes of incubation in the CRISPR step. **(c)** Bar chart of the fluorescence endpoint values for the clinical sample and negative control after 20 minutes of incubation in the CRISPR step. **(d)** Sequencing results of the RPA amplification product for the clinical sample. **(e)** Alignment of the base sequence obtained from sequencing of the clinical sample with the NCBI database. The comprehensive results indicate a high similarity between the RPA amplification product sequence of the sample and various HPV 35 sequences in the NCBI database.


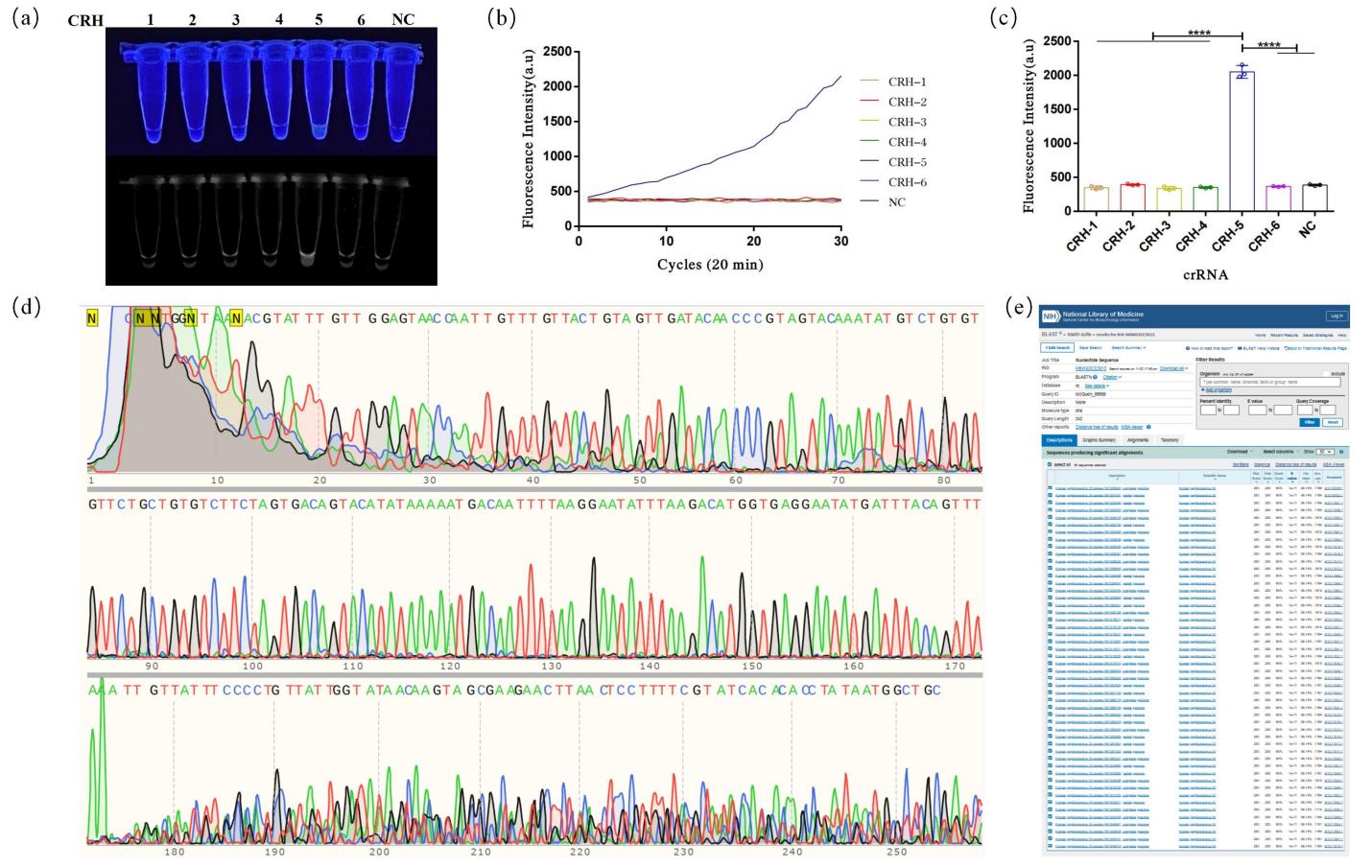


Figure S3. QPCR amplification curves for eight clinical samples positive for HPV 16, labeled as S16-1 to S16-8.


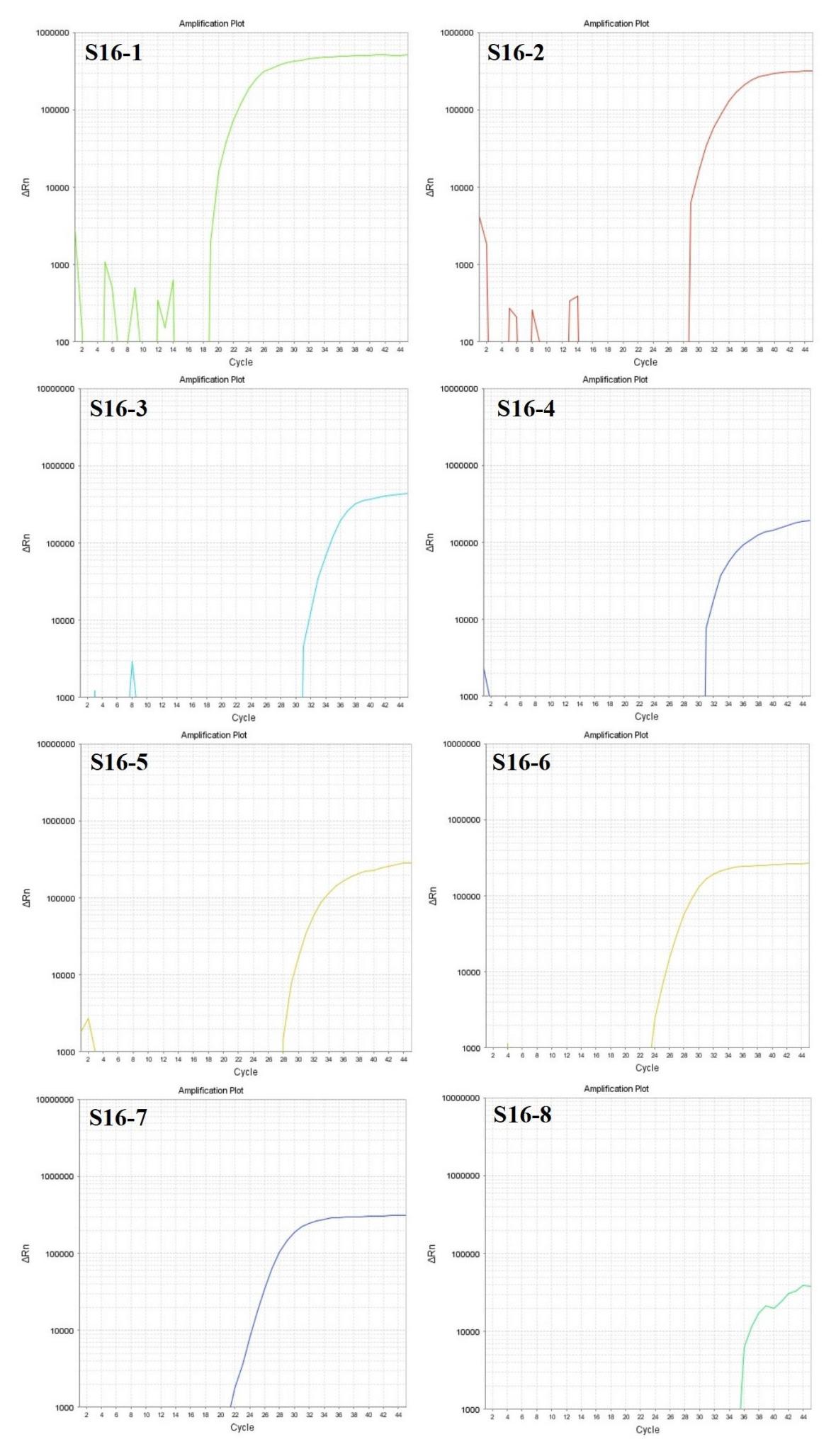


Figure S4. QPCR amplification curves for eight clinical samples positive for HPV 18, labeled as S18-1 to S18-8.


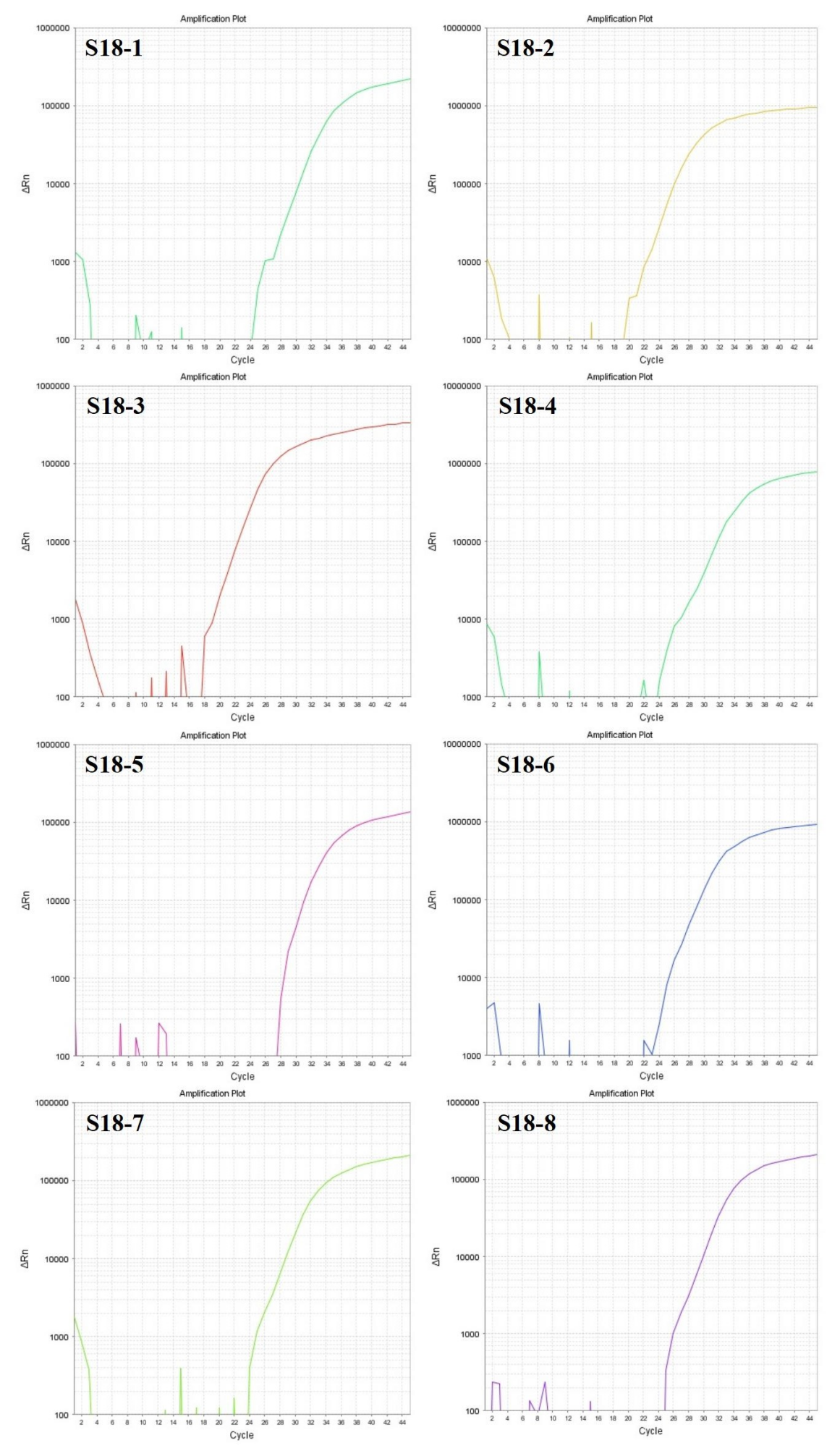


Figure S5. QPCR amplification curves for three clinical samples positive for HPV 31, labeled as S31-1 to S31-3, and for HPV 33, 35, and 31/45 double-positive clinical samples labeled as S33-1, S35-1, and S31/45, respectively.


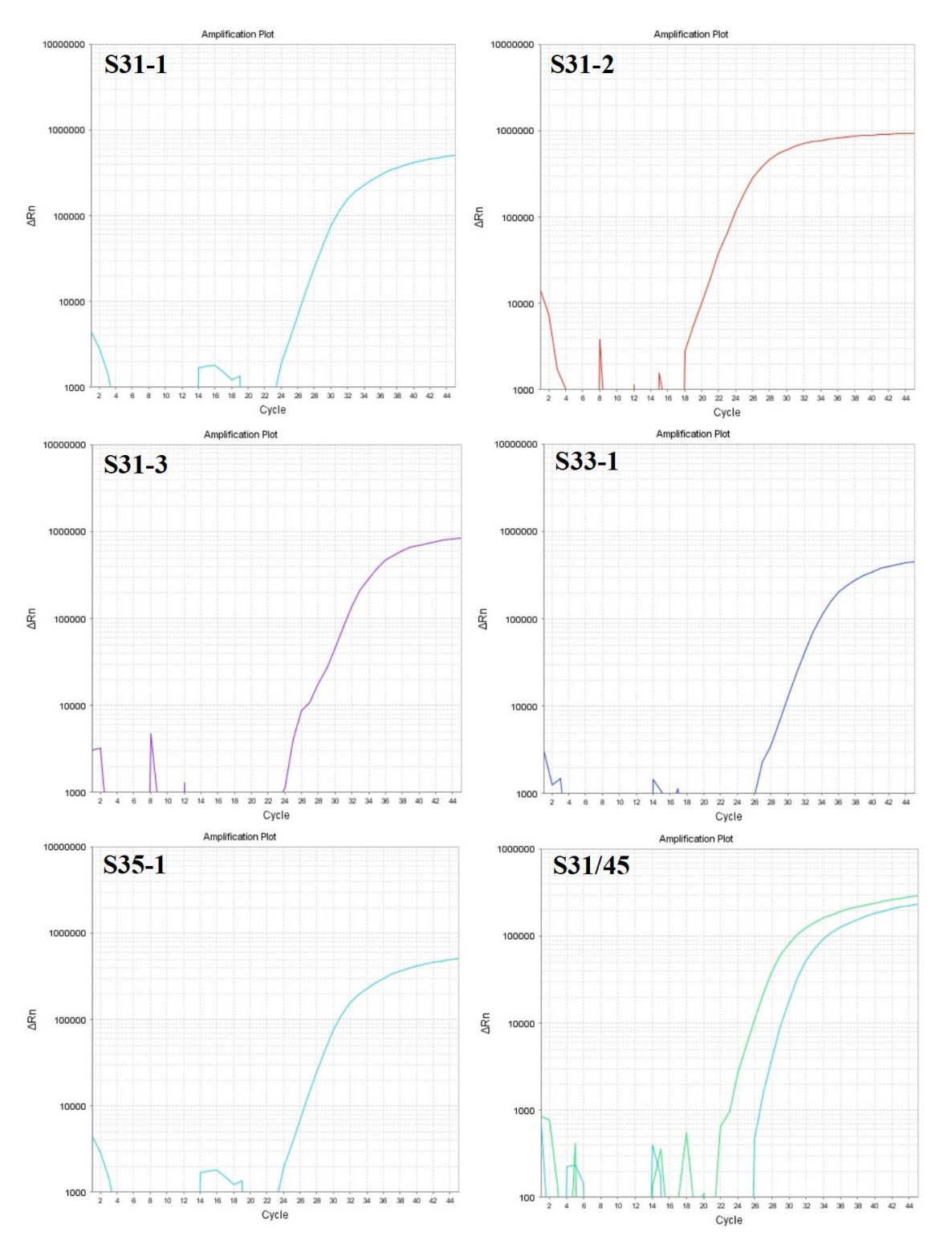


Figure S6. QPCR amplification curves for clinical samples positive for various additional high/low-risk HPV types, detected by the H-MRC12a method. **S39/53**: HPV 31/53 double-positive sample. **S51/52**: HPV 51/52 double-positive sample. **S58/6**: HPV 58/6 double-positive sample. **S68, S81, S82**: Samples positive for HPV 68, 81, and 82, respectively.


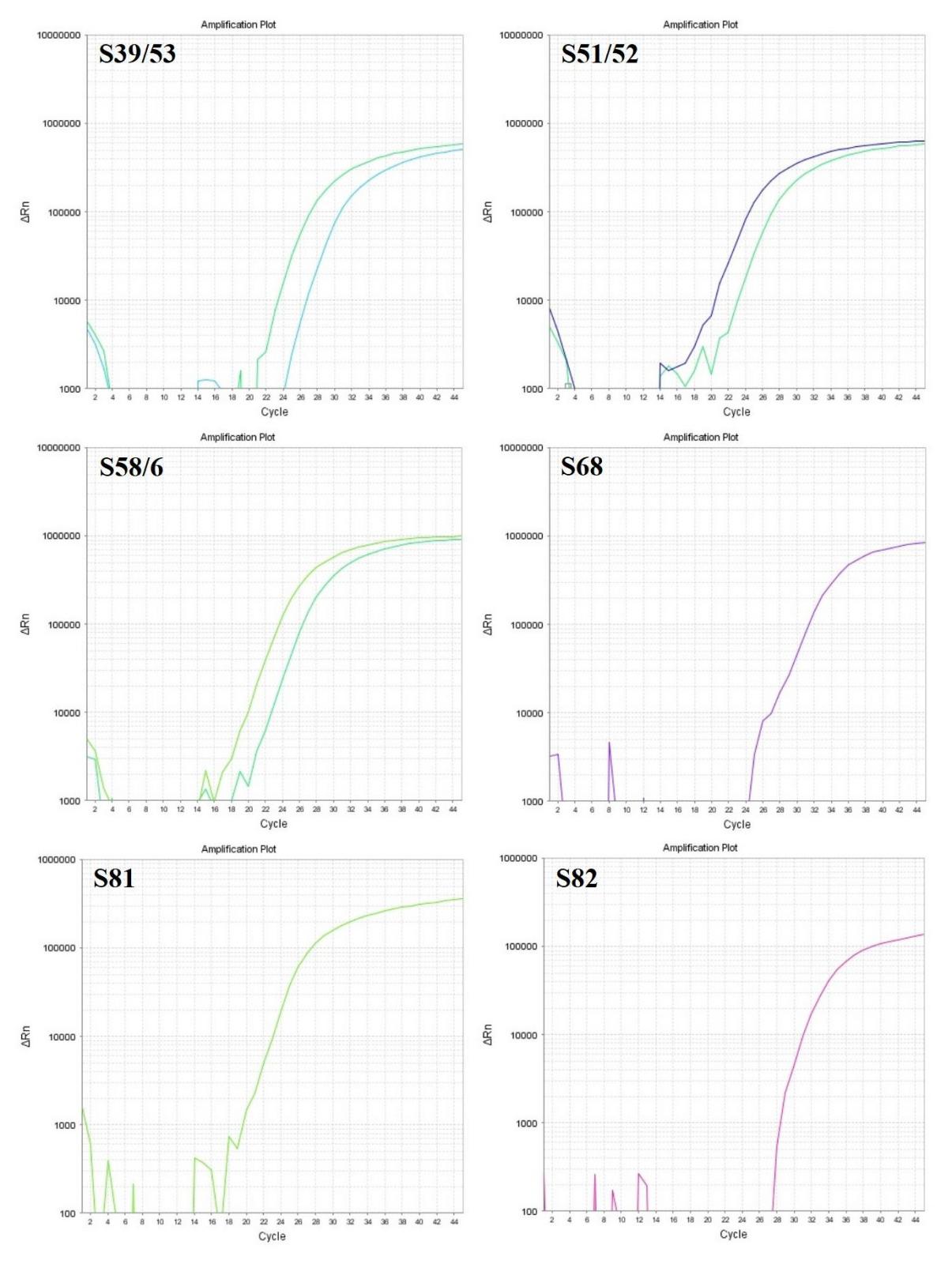

Supplement: Supplementary file 1 — Additional file 1: Table S1. Sequences of plasmids. Table S2. Sequences of primers. Table S3. Coverage of HPV 16/18/31/33/35/45 crRNAs used for the H-MRC12a. Figure S1. H-MRC12a method detection results and sequencing alignment of the RPA amplification product for an HPV 31-positive clinical sample not detected by the QPCR method. a The sample, after heat inactivation, underwent nucleic acid extraction using the nanomagnetic bead method. The extracted nucleic acids were incubated in the H-MRC12a combined detection system for 40 mins (Multiple RPA incubation for 20 mins, followed by CRISPR incubation for an additional 20 mins) and immediately observed under 300 nm UV light. b Real-time fluorescence signals (recorded using the Q160 LongGene portable QPCR instrument) of the clinical sample and negative control after 20 mins of incubation in the CRISPR step. c Bar chart of the fluorescence endpoint values for the clinical sample and negative control after 20 mins of incubation in the CRISPR step. d Sequencing results of the RPA amplification product for the clinical sample. e Alignment of the base sequence obtained from sequencing of the clinical sample with the NCBI database. The comprehensive results indicate a high similarity between the RPA amplification product sequence of the sample and various HPV 31 sequences in the NCBI database. Figure S2. H-MRC12a method detection results and sequencing alignment of the RPA amplification product for an HPV 35-positive clinical sample not detected by the QPCR method. a The sample, after heat inactivation, underwent nucleic acid extraction using the nanomagnetic bead method. The extracted nucleic acids were incubated in the H-MRC12a combined detection system for 40 mins (Multiple RPA incubation for 20 mins, followed by CRISPR incubation for an additional 20 mins) and immediately observed under 300 nm UV light. b Real-time fluorescence signals (recorded using the Q160 LongGene portable QPCR instrument) of the clinical sample and [file 11658_2024_548_MOESM1_ESM.doc]
